# Strengths of covalent bonds in LnO_2_ determined from O K-edge XANES spectra using a Hubbard model†

**DOI:** 10.1039/d3sc03304j

**Published:** 2023-11-03

**Authors:** Wayne W. Lukens, Stefan G. Minasian, Corwin H. Booth

**Affiliations:** a Chemical Sciences Division, Lawrence Berkeley National Laboratory Berkeley CA 94720 USA wwlukens@lbl.gov

## Abstract

In LnO_2_ (Ln = Ce, Pr, and Tb), the amount of Ln 4f mixing with O 2p orbitals was determined by O K-edge X-ray absorption near edge (XANES) spectroscopy and was similar to the amount of mixing between the Ln 5d and O 2p orbitals. This similarity was unexpected since the 4f orbitals are generally perceived to be “core-like” and can only weakly stabilize ligand orbitals through covalent interactions. While the degree of orbital mixing seems incompatible with this view, orbital mixing alone does not determine the degree of stabilization provided by a covalent interaction. We used a Hubbard model to determine this stabilization from the energies of the O 2p to 4f, 5d(e_g_), and 5d(t_2g_) excited charge-transfer states and the amount of excited state character mixed into the ground state, which was determined using Ln L_3_-edge and O K-edge XANES spectroscopy. The largest amount of stabilization due to mixing between the Ln 4f and O 2p orbitals was 1.6(1) eV in CeO_2_. While this energy is substantial, the stabilization provided by mixing between the Ln 5d and O 2p orbitals was an order of magnitude greater consistent with the perception that covalent bonding in the lanthanides is largely driven by the 5d orbitals rather than the 4f orbitals.

## Abstract 2

A Hubbard model allows the covalent contribution to bond strength to be determined spectroscopically from O K-edge X-ray absorption fine structure spectra.

## Graphical abstract

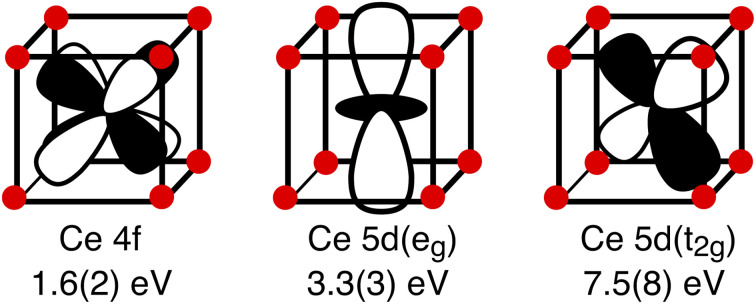

## Introduction

Bonding in lanthanide (Ln) complexes has traditionally been viewed as largely ionic due in large part to the core-like nature of the 4f orbitals.^1^ Nevertheless, work by Axe, Jørgensen, and others in the 1960s demonstrated that the splitting of the 4f orbitals is largely due to overlap between the 4f orbitals and ligand orbitals rather than electrostatic effects.^2,3^ More recently, Alessandri *et al.*, Ungur, and Chibotaru reached similar conclusions based on *ab initio* calculations.^4,5^ In other words, while electrostatic effects play a larger role than orbital overlap in determining the stabilities of lanthanide compounds, splitting of the 4f orbitals is largely due to orbital overlap rather than electrostatic effects. The splitting of the 4f orbitals is small in trivalent Ln complexes, 10^2^ to 10^3^ cm^−1^,^6,7^ which is consistent with the view that mixing between the 4f and ligand orbitals contributes little to bond strength in Ln compounds. This observation lead to the FEUDAL (f's essentially unaffected; d's accommodate electrons) model for bonding in the trivalent lanthanides and actinides (Ln and An).^8^

In the 1980s and 1990s, the degree of Ln 4f and O 2p orbital mixing (covalency) in CeO_2_, PrO_2_, and TbO_2_ was quantified by modeling Ln L_3_-edge X-ray absorption near edge structure (XANES) spectra.^9–11^ The XANES and photoemission spectra of CeO_2_ have been extensively studied and modeled using an Anderson impurity model that includes mixing between the Ce 4f and O 2p orbitals; the results support the importance of this mixing on the spectroscopic properties of CeO_2_.^10,12–15^ For CeO_2_, the degree of mixing determined from its XANES spectrum was supported by core X-ray photoelectron spectroscopy performed by Fujimori.^16^ High pressure Ce L_3_-edge measurements by Kaindl *et al.* further buttressed the role of Ce 4f and O 2p mixing in CeO_2_.^17^

In 2017, Minasian *et al.*, reported the O K-edge spectra of LnO_2_.^18^ As originally demonstrated at the Cl K-edge by Shadle *et al.*, ligand K-edge spectra provide a quantitative measurement of the mixing between the ligand and metal orbitals.^19,20^ The O K-edge measurements showed several intense and well-resolved transitions, which were attributed to mixing between O 2p orbitals and the Ln 4f, 5d(t_2g_) and 5d(e_g_) orbitals. Surprisingly, the amount of orbital mixing between the O 2p and 4f orbitals was comparable to that between the O 2p and 5d orbitals, which challenges the perception that the 4f orbitals do not contribute strongly to bonding in LnO_2_. However, the XANES spectra alone do not allow one to determine how much of the LnO_2_ lattice strength is due to covalent bonding resulting from mixing between the O 2p and Ln 4f orbitals.

In contrast to the covalent interactions, the ionic contribution to bonding in LnO_2_ can be estimated from their Madelung energies, which were calculated by Angelow from the lattice parameters.^21^ Angelow also calculated the lattice stabilization (bond strength for an extended solid) using a Born–Haber cycle and measured thermodynamic values.^21^ The lattice stabilization was found to be very similar to the Madelung energy in LnO_2_. Therefore, ionic bonding contributes far more stabilization than covalent bonding involving either the Ln 4f or 5d orbitals. This example underscores an asymmetry between estimating the strengths of ionic interactions, for which approaches based on the Madelung energy exist,^22–25^ and estimating the strengths of covalent interactions, which primarily rely on calculations, especially energy decomposition analysis.^26–33^ While it is possible to estimate the ionic contribution to bonding from structural parameters, no analogous approach for quantifying the covalent contribution to bonding based on physical measurements is widely used.

The evolution of the description of bonding in Ln compounds from wholly ionic to weakly covalent mirrors the evolution of the understanding of bonding in transition metal complexes, which was also once thought to be almost wholly ionic. In 1966, Hubbard, Rimmer, and Hopgood presented a theory for transition metal bonding based on configuration interactions (CI) that mix excited state character into the ground state as an alternative to molecular orbital theory (MO), which focuses on orbital mixing.^34^ These theories are equivalent when CI is included in MO theory, but Hubbard's CI model is rarely used. The original intent of Hubbard *et al.* was to use the CI model to calculate the wavefunctions and properties of transition metal complexes. This was only partially successful, presumably due to limited computational capabilities in 1966. Nevertheless, a simplified CI model based on valence bond theory (VB) states can be used to quantify the covalent contribution to bonding from two experimentally determined parameters: the energy of the charge-transfer (CT) transition related to a specific orbital interaction (*e.g.*, between the Ln 4f orbitals and O 2p orbitals) and the amount of excited CT state character mixed into the ground state by the CI associated with this interaction. The CT energy may be obtained by optical spectroscopy. In the case of CeO_2_, the O 2p to Ce 4f CT band gap is 3.23(5) eV as measured by diffuse reflectance.^35^ The amount of this excited state character that is mixed into the ground state can be measured by Ce L_3_-edge XANES. It was found to be 0.5 by Dexpert *et al.*,^8^ 0.54 by Bianconi *et al.*,^10^ and later determined to be 0.56(4) by Minasian *et al.*^9,18^ More generally, the mixing has been determined from the ligand K-edge.^20^ The attraction of Hubbard's CI model is that it provides a way to determine the covalent contribution to bond strength from experimental measurements analogously to the way in which the ionic contribution bay be estimated from the Madelung energy.

In this paper we report a simplified CI model (Hubbard Molecule Model, HMM), which can be used to determine covalent contributions to bond strength. This model has been used to determine the strength of 4f-ligand bonds in (C_8_H_8_)_2_Ce and 
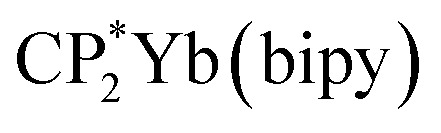
, where C_8_H_8_ is cyclooctatetraene, Cp* is pentamethylcyclopentadienyl, and bipy is 2,2′-bipyridyl.^36,37^ In this paper, we extend the model to interactions with spatial degeneracy, show that the Wolfsberg–Helmholz approximation^38^ can be used to estimate the Hubbard hopping integral, *t*, and develop a second-order version of the HMM. We report the O 2p to Ln 4f CT band gap energies for PrO_2_ and TbO_2_ determined from their diffuse reflectance spectra, and we report the O 2p to Ce 5d(e_g_) and 5d(t_2g_) band gap energies, which were determined from the diffuse reflectance spectrum of CeO_2_ from 0.5 eV to 20 eV measured by Niwano *et al.*^39^ We show that the energies of the O K-edge XANES pre-edge peaks are related to the energies of the CT bands by a simple energy offset. Using this relationship, we obtain the average CT band energies from the energies of the O K-edge pre-edge peaks. Finally, we use this information and the HMM to determine the contributions of the 4f and 5d orbitals to bonding. These covalent contributions to bonding are compared to the ionic contribution estimated using Bohr–Landé theory.

## Experimental

PrO_2_ and TbO_2_ were prepared as previously described.^18,40^ CeO_2_ for magnetic susceptibility measurements was purchased (Strem PURATREM 58-0800, 99.995% CeO_2_; Aldrich 202 975, 99.995% CeO_2_), dried under vacuum for 48 h to remove adsorbed water, and stored under argon in an inert atmosphere glovebox. Quartz wool (Hereaus, electronic grade) was leached with 0.1 M oxalic acid in 0.5 M sulfuric acid, washed with DI water and dried at 120 °C in air then dried under vacuum for 48 h before use.^41^ Detailed descriptions of magnetic susceptibility measurements, diffuse reflectance spectroscopy, and determining band gap energies using a Tauc plot are given in the ESI.†

## Results and discussion

### Hubbard molecule model (HMM)

The HMM is a simplified version of the model originally described by Hubbard *et al.* for transition metal compounds.^34^ The main difference is that the original model included all of the electrons and interactions in the system, but the HMM only includes a single interaction (*e.g.*, that between the Ln 5f t_2g_ orbitals and the corresponding O 2p orbitals). In addition, the HMM is based on the valence bond (VB) description of the states corresponding to that interaction. The HMM is a “toy model”, which describes a single metal site rather than an extended solid. The distinction is necessary to avoid confusion between the HMM and the better-known Hubbard Model for extended solids.^42^

One simplification of the HMM relative to the model described by Hubbard *et al.* is that the basis set for the HMM consists of the valence bonding (VB) states of the molecule (LnO_8_^12−^ site in LnO_2_ in this case) with the atoms in the positions they are found in the molecule or extended solid. The energies of the VB basis states include the effects of electrostatic interactions and spin–orbit coupling but do not include the stabilization due to orbital overlap (see eqn (3.1) in ref. 34). In the HMM, the ground state is stabilized by CI with excited CT states, which is analogous to stabilization of the bonding orbital in an MO model due to orbital overlap. The interaction energy between the ground state and the charge-transfer state is given by the “hopping integral,” *t*. The magnitude of *t* is related to both the overlap between the states and the absolute energies of the states in analogy to the orbital interaction energy or off-diagonal matrix element (H_12_) in an MO model.^43^ In the HMM, *t* is identical to H_12_ but with the opposite sign due to the different conventions used in constructing the HMM Hamiltonian and the MO Hamiltonian. The magnitude of *t* is related to *V*_kf_ in the Anderson impurity model,^13^ which unlike the HMM is not a “toy” model and includes more detail about the electronic structures of these systems. The energy of the excited VB basis state relative to the ground VB basis state is given by *U*′, which is related to the difference in energies between the basis orbitals in an MO model. However, *U*′ also includes the effects of electron correlation, primarily electron repulsion due to pairing. The meanings of *t* and *U*′ in the HMM are best illustrated by the example given below.

### HMM for 2 electrons in 2 orbitals – 4f orbital interaction in CeO_2_ and PrO_2_

**Fig. 1:**
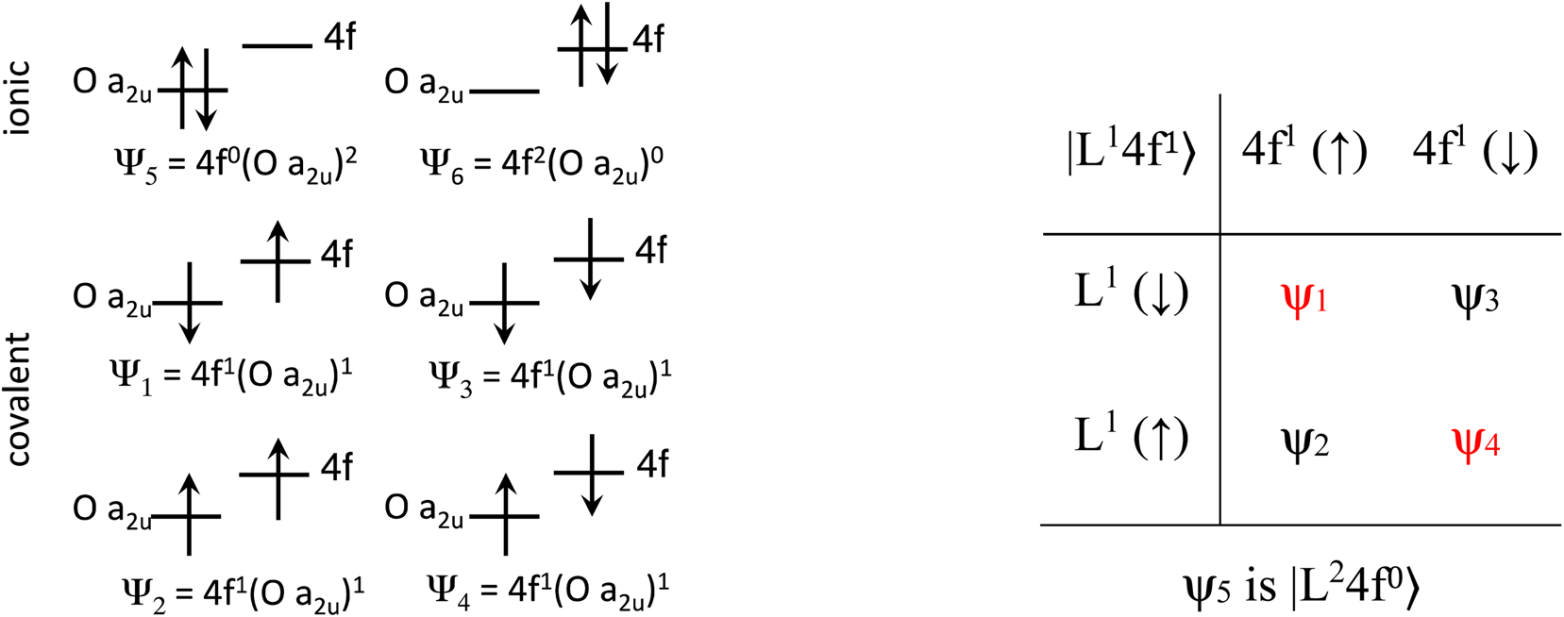
Basis states for the 4f interaction in CeO_2_(left). Compact representation (right).

The Ln site in LnO_2_ has *O*_h_ symmetry and consists of an Ln at the center of a cube with oxygen located at the vertices. From MO theory, a single Ce 4f orbital, 4f_*xyz*_, with A_2u_ symmetry, strongly interacts with the oxygen atoms; the Ce 4f orbital t_1u_ has smaller overlap.^18^ The relatively strong interaction for a 4f orbital is due to the fact that the lobes of the 4f_*xyz*_ orbital point directly at the oxygen atoms of CeO_2_. The HMM includes the Ce 4f_*xyz*_ orbital and the corresponding symmetry adapted linear combination (SALC) of the O 2p orbitals. From Ce L_3_ XANES measurements, the amount of 4f electron character donated from the O^2−^ ligands to Ce^4+^, *n*_f_, is 0.56(4).^9,18^ Since *n*_f_ is greater than 0.5, the ground VB state for CeO_2_ in the HMM consists of an unpaired electron on the Ce center and an unpaired electron in the corresponding O 2p orbital. If *n*_f_ were less than 0.5, the ground VB state would have no unpaired electrons on Ce and a pair of electrons in the corresponding O 2p orbital. For Ce, the effect of spin–orbit coupling on the degeneracy of the state must be included. The Ce 4f_*xyz*_^1^ state involved in the interaction largely contributes to the *Γ*_7_ state when spin–orbit coupling is included. Like 4f_*xyz*_, *Γ*_7_ has only one degree of spatial degeneracy. The basis states for the 4f orbital interaction in CeO_2_ are shown in Fig. 1. The states can be more compactly written using Dirac notation (Fig. 1, right). In the basis states, the effect of overlap between the O 2p and Ce 4f orbitals is not included, so the singlet ground VB states *Ψ*_1_ and *Ψ*_4_, |L^1^4f^1^〉 in Dirac notation, are energetically degenerate. The triplet VB basis states, *Ψ*_2_ and *Ψ*_3_, are lower in energy than *Ψ*_1_ and *Ψ*_4_ due to the 〈*Φ*_Ce_(1)*Φ*_O_(2)|1/*r*_12_|*Φ*_Ce_(2)*Φ*_O_(1)〉 term in the exchange integral.^44,45^*Ψ*_1_–*Ψ*_4_ are covalent in the VB nomenclature because the Ce f-orbital and O 2p SALC are each occupied by a single electron. Excited VB state *Ψ*_5_, |L^2^4f^0^〉, is higher in energy by *U*′ and is ionic in the VB sense since both electrons are in the O 2p orbital. State *Ψ*_6_, |L^0^4f^2^〉, which is also ionic, corresponds to Ce(ii) and is much higher in energy. This state can contribute to bonding in other systems, especially those involving lanthanides with easily accessible divalent states (Eu, Sm, and Yb).^46^ In LnO_2_, we assume that the contribution from |L^0^4f^2^〉 is negligible, which is consistent with the *ab initio* calculations by Sergentu *et al.*^46^ In the HMM, only states with the same spin can interact (only *Ψ*_1_ and *Ψ*_4_ can interact with *Ψ*_5_), so the triplet states are omitted. The ground state in the HMM is stabilized by CI between |L^1^4f^1^〉 and |L^2^4f^0^〉.

The HMM Hamiltonian for the interaction between the |L^1^4f^1^〉 and |L^2^4f^0^〉 in CeO_2_ can be described using the matrix shown in eqn (1). Since the model only involves differences in energy between the states, the energy of the |L^1^ 4f^1^〉 ground state is set to zero.1
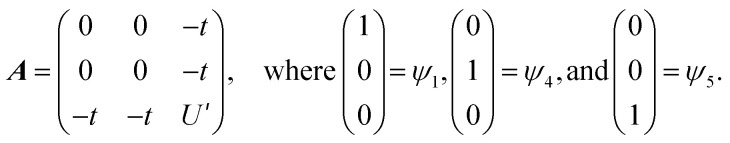


**Fig. 2:**
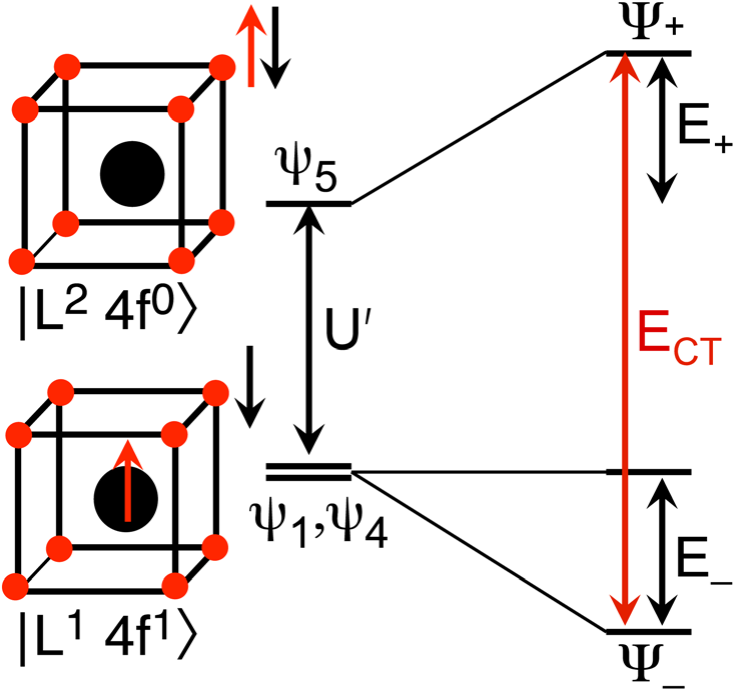
HMM for the 4f interaction in CeO_2_ and PrO_2_.

To first order, the energies and wavefunctions for the HMM can be determined from |***A*** − *E****I***| = 0, where ***I*** is the identity matrix. The energies are 0 and 
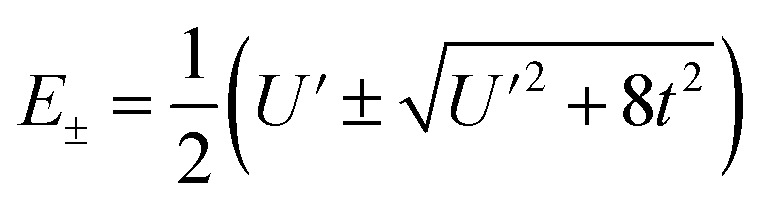
, and the states of interest are *Ψ*_±_, which have energies *E*_±_ and are given by *Ψ*_−_ = *N*(|L^1^ 4f^1^〉 + *λ*|L^2^ 4f^0^〉) and *Ψ*_+_ = *N*(|L^2^ 4f^0^〉 + *λ*|L^1^ 4f^1^〉) where 
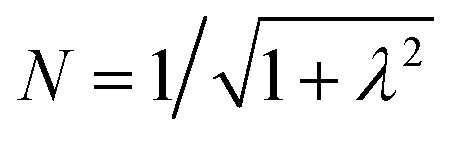
 and 
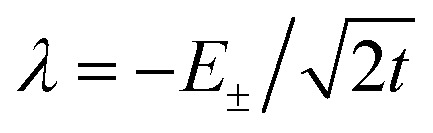
. In the HMM of CeO_2_, the ground state is *Ψ*_−_, which is a linear combination of the singlet |L^1^ 4f^1^〉 states that are stabilized by CI with the charge-transfer state |L^2^ 4f^0^〉. The HMM for the 4f orbital interactions in CeO_2_ is illustrated in Fig. 2.

Because the HMM is based on states, certain energies can be determined spectroscopically. The difference in energy between the ground state, *Ψ*_−_, and the destabilized state, *Ψ*_+_, is the energy of a charge-transfer band, *E*_CT_, which is equal to 
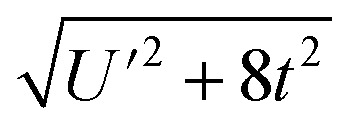
. The amount of 4f electron density in the ground state can also be measured spectroscopically using either Ln L_3_-edge or O K-edge XANES spectroscopy.^9,18^ In this example, the 4f electron density, *n*_f_, is equal to *N*^2^, where *N* is the normalization constant for *Ψ*_−_. It is not necessary to determine *t* and *U*′ to calculate *E*_−_; as shown by Hubbard, the stabilization to first order, is the product of the amount of excited state character mixed into the ground state and the charge-transfer energy, which is (1 − *n*_f_) × *E*_CT_ in this case.^34^

Examples of the application of the HMM to interactions between the O 2p and Ln 5d orbitals are provided in the ESI.† The main differences are that the ground states are ionic in the VB sense and that the degeneracy of the states must be taken into account when comparing *N*^2^ to the amount of excited state O 2p to Ln 5d CT character, *n*_d_, mixed into the ground state.

### Quantifying excited state character mixed into the ground state (*n*_f_ and *n*_d_) in LnO_2_

Our primary goal is to determine how much covalent interactions involving each orbital, 4f, 5d(e_g_), and 5d(t_2g_), contribute to the bond strength of LnO_2_, which is given by *E*_−_ for each orbital. To determine this energy, we need to know both the energies of the excited CT states, *E*_CT_, and the amount of electron density that is transferred by the CI, which is related to the values of *n*_f_ or *n*_d_, associated with those excited states. The amount of electron density, *n*_f_, in the 4f orbitals can be measured using Ln L_3_-edge XANES spectroscopy as initially shown by Dexpert *et al.*^9,10,47^ More generally, Minasian *et al.*, demonstrated that the amount electron density in the 4f, 5d(t_2g_), and 5d(e_g_) orbitals of LnO_2_ can be quantified by O K-edge XANES spectroscopy.^18^ The electron density transferred from O to Ln in LnO_2_ is given in Table 1. For CeO_2_ and TbO_2_, the values of *n*_f_ were determined by fitting the Ln L_3_-edge XANES spectra as previously reported, and for PrO_2_, the value of *n*_f_ was determined from the O K-edge XANES pre-edge peak assigned to the 4f a_2u_ interaction.^18^ The values of *n*_d_(e_g_) and *n*_d_(t_2g_) were determined from the normalized areas of the O K-edge XANES pre-edge peaks using the I_1s–2p_ transition element determined by Minasian *et al.*^18^

**Table 1:** Total amount of excited state character, *n*_f_ and *n*_d_, mixed into the ground states of LnO_2_ determined from ref. 18. The uncertainty is given in parentheses in the same units as the preceding digit

	CeO_2_	PrO_2_	TbO_2_
*n*_f_	0.56(4)	0.53(5)	0.42(4)
*n*_d_(e_g_)	0.59(6)	0.63(6)	0.59(6)
*n*_d_(t_2g_)	1.05(12)	1.12(14)	1.05(12)

### Energies of the CT states in LnO_2_

Unlike the values of *n*_f_ and *n*_d_, the CT band energies, *E*_CT_, have not been reported for all LnO_2_. We would like to know the average energy of the band; however, the energy that is most readily determined is the band gap (*E*_BG_), which corresponds to the lowest energy CT states in the band and can be determined using a Tauc plot.^48,49^ While the band edge, from which the band gap is determined, is generally obvious in diffuse reflectance (DR) spectra, the center of the band, from which the average energy may be determined, is not necessarily obvious. In contrast to the DR spectra, the O K-edge XANES pre-edge peaks are well-resolved, and their average energies, which are closely related to the average band energies, may be obtained by fitting to the spectra. Therefore, our goal is to measure the band gaps of the LnO_2_ compounds by DR and compare them to the corresponding band gaps determined from the O K-edge pre-edge peaks as previously done for MO_4_^*n*−^ (M = Cr, Mo, W, Mn, Re, Tc, and *n* = 1 or 2).^50^ By comparing these energies, the relationship between the CT energy and the energies of the O K-edge pre-edge peaks can be determined, which will allow us to determine the average energies of the CT bands from the O K-edge pre-edge peaks.

**Fig. 3:**
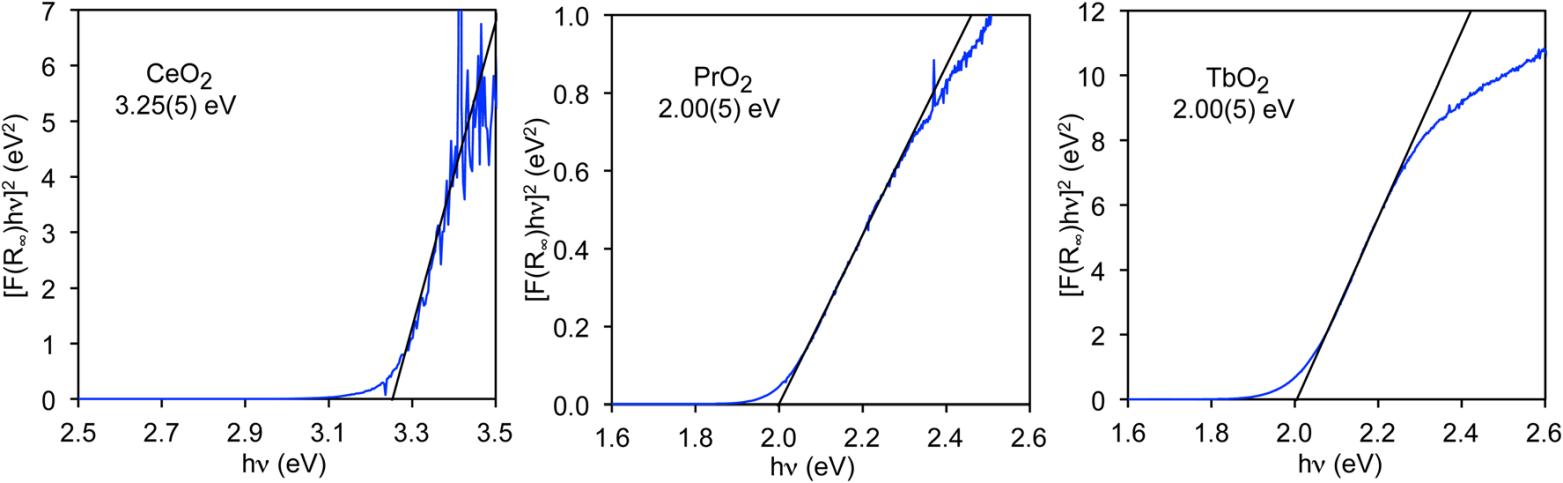
Tauc plots for CeO_2_, PrO_2_, and TbO_2_. Band gaps are given below the chemical formulae.

The value of *E*_BG_ for the O 2p to Ce 4f CT band in CeO_2_, *E*_BG_(4f), has been determined for both bulk CeO_2_ and nanoparticles using Tauc plots.^48^ For bulk material, *E*_BG_(4f) is 3.23(5) eV.^35^ The 4f band gaps in PrO_2_ and TbO_2_ have not previously been reported, but can also be determined from Tauc plots as shown in Fig. 3. The value we obtain for CeO_2_, 3.25(5) eV, is included to illustrate that we obtain the reported value within error. In both PrO_2_ and TbO_2_, *E*_BG_(4f) is 2.00(5) eV.^51^ The values of *E*_BG_(4f) in PrO_2_ and TbO_2_ were previously calculated to be 2.3 eV and 1.7 eV, respectively, which are in reasonable agreement with our measurements.^18^

The energies of *E*_BG_(5d(e_g_)) and *E*_BG_(5d(t_2g_)) in CeO_2_ were determined to be 6.6(1) eV and 9.0(1) eV, respectively, from the DR spectrum of CeO_2_, which was measured from 4 to 20 eV by Niwano *et al.*^39^ The value of *E*_BG_(4f) was also determined from the data of Niwano *et al.*, and found to be 3.3(1) eV, which is slightly larger than the values reported by others and measured by us. For this reason, the uncertainties in the values of the band gaps determined from this spectrum are estimated to be 0.1 eV (Tauc plots are given in Fig. S3†).

**Fig. 4:**
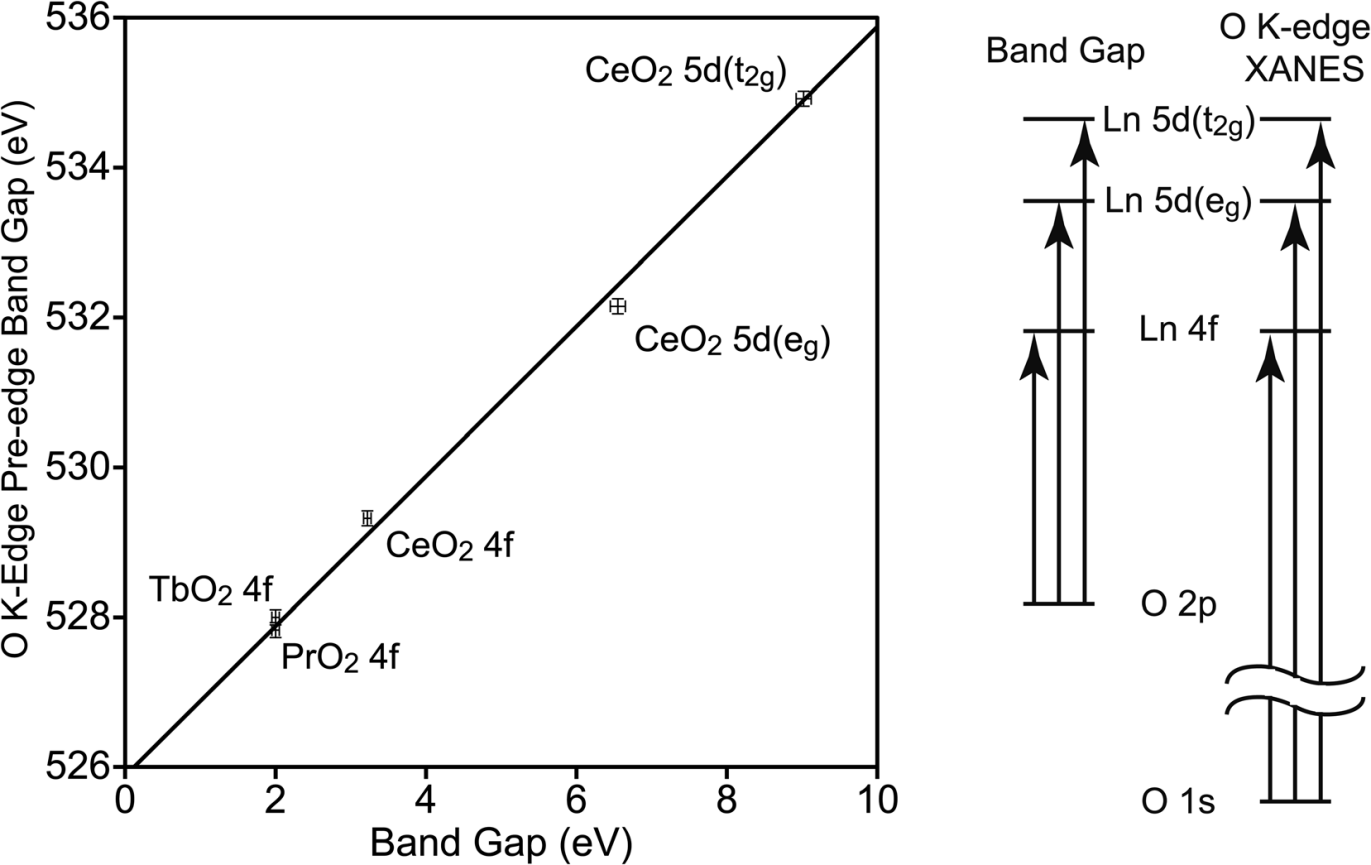
Relationship between the O K-edge pre-edge peak band gaps and band gaps in LnO_2_ (left). Uncertainties are illustrated by error bars. The line, *E*_PE-BG_ = *E*_BG_ + 525.9(1) eV, is fit to the data. Transitions related to these energies (right).

To determine the relationship between *E*_BG_ and the band gaps of the O K-edge XANES pre-edge peaks (*E*_PE-BG_), the latter were determined using Tauc plots (Fig. S4–S6†). The values of *E*_BG_ and *E*_PE-BG_ are closely related, and this relationship has been used when examining the electronic structure of metal oxides.^50,52^ The CT states involve a transition from an O 2p orbital to a Ln 4f or 5d orbital, leaving a hole in the O 2p orbitals and adding an electron to a Ln 4f or 5d orbital. In the O K-edge XANES pre-edge peaks, the hole is in the O 1s orbital, and the electron has been excited into the same 4f or 5d orbital (see Fig. 4). The difference between the *E*_BG_ and *E*_PE-BG_ is the difference between the energies of the O 1s and O 2p orbitals in the O^2−^ ligand in LnO_2_, which should be similar to the energy of the O 1s to O 2p transition in atomic oxygen, 527.9 eV.^53^ The energy will not be identical since O^2−^ has two additional electrons relative to atomic O, and since the electrons of O^2−^ are stabilized by the Ln^4+^ center. The energies of the XANES pre-edge peak band gaps are plotted against the energies of the CT band gaps in Fig. 4. This data can be fit using a simple energy offset of 525.9(1) eV with a reduced chi-squared (*χ*_*ν*_^2^) of 2.5. The data was also fit using a linear model, *E*_PE-BG_ = *b*_0_ + *b*_1_*E*_BG_, *b*_0_ is 526.0(1) eV and *b*_1_ is 0.98(2) with a slightly larger *χ*_*ν*_^2^ of 2.8. For this data, the simple model with an energy offset of 525.9(1) eV between the O K-edge XANES pre-edge features and the CT peaks better fits the data. As expected, the energy offset is close to the energy of the 1s to 2p transition in atomic oxygen; it is also similar to the value of 526.9(1) eV observed in MO_4_^*n*−^.^50^

The energy offset of 525.9(1) eV of *E*_PE-BG_ relative to *E*_BG_ allows the average energies of the CT bands (*E*_CT_) to be determined from the average energies of the O K-edge pre-edge peaks, which were reported by Minasian *et al.*^18^ The values of *E*_CT_ determined in this way are given in Table 2. The values in Table 2 may be compared to those determined by other techniques. The value of *E*_CT_(4f) has been determined from DR to be 3.9 eV and 4 eV by Marabelli and Wachter and by Niwano *et al.*, respectively.^39,54^ The value we obtain from the O K-edge spectrum, 4.30(14) eV, is slightly larger. In CeO_2_ the difference in energy between 5d(e_g_) and 5d(t_2g_) is 3.6(3) eV. This value may be compared to the difference in energy of these states determined at the Ce L_3_-edge, which is 4.0 eV.^55^ However, the energy difference at the Ce L_3_-edge is increased due to the presence of the Ce 2p core-hole, which is estimated to increase the energy difference by 0.5 eV.^15^ Taking this increase into account, the difference in energy between 5d(e_g_) and 5d(t_2g_) in CeO_2_ is estimated to be 3.5 eV from the Ce L_3_-edge XANES spectrum, which is in good agreement with the value of 3.6(3) eV that we obtain from the O K-edge XANES spectrum of CeO_2_.

**Table 2:** Average energies of charge-transfer bands, *E*_CT_, in LnO_2_ in eV as determined from the energies of the O K-edge pre-edge peaks. The uncertainty is given in parentheses in the same units as the last digit

*E*_CT_	CeO_2_	PrO_2_	TbO_2_
4f	4.30(14)	2.90(14)	2.90(14)
5d(e_g_)	7.10(14)	7.10(14)	6.80(14)
5d(t_2g_)	10.70(14)	10.70(14)	10.30(14)

### First order HMM for LnO_2_

Using the data in Tables 1 and 2, the values of *t*, *U*′, and *E*_−_ can be determined in LnO_2_ using the HMM. The results are given in Table 3. As shown in the next section, these stabilization energies have a systematic error due to using a first order model. The implications of the values of the parameters are considered in the Discussion section.

**Table 3:** Stabilization of LnO_2_ by 4f and 5d interactions with O 2p orbitals to first order. Values in eV. The uncertainty is given in parentheses in the same units as the last digit

	CeO_2_	PrO_2_	TbO_2_
*t* 4f	1.51(5)	1.02(5)	0.84(5)
*U*′ 4f	0.5(3)	0.2(3)	1.7(1)
*E*_−_ 4f	−1.9(2)	−1.4(2)	−0.61(7)
Total 4f stabilization^a^	−1.9(2)	−1.4(2)	−1.2(1)
*t* 5d(e_g_)	2.29(8)	2.33(8)	2.19(8)
*U*′ 5d(e_g_)	2.9(4)	2.6(4)	2.8(4)
*E*_−_ 5d(e_g_)	−2.1(2)	−2.2(2)	−2.0(2)
Total 5d(e_g_) stabilization^a^	−4.2(4)	−4.5(4)	−4.0(4)
*t* 5d(t_2g_)	3.6(1)	3.7(1)	3.5(1)
*U*′ 5d(t_2g_)	3.2(9)	3(1)	3.1(8)
*E*_−_ 5d(t_2g_)	−3.7(4)	−4.0(5)	−3.6(4)
Total 5d(t_2g_) stabilization^a^	−11(1)	−12(2)	−11(1)

a Total stabilization includes the degeneracy of the state.

### Second order HMM for LnO_2_

Since the stabilization due to orbital overlap (*E*_−_ and total stabilization in Table 3) were determined to first order in the HMM, these energies are larger than actual stabilization. The energies can be determined to second order using the secular equation (eqn (2)) rather than using the usual Hubbard model solution, which assumes *S* = 0 in eqn (2). For the CeO_2_ 4f interaction, the determinant is given in eqn (2), where *S* is the group overlap between the O 2p and Ce 4f orbitals.2
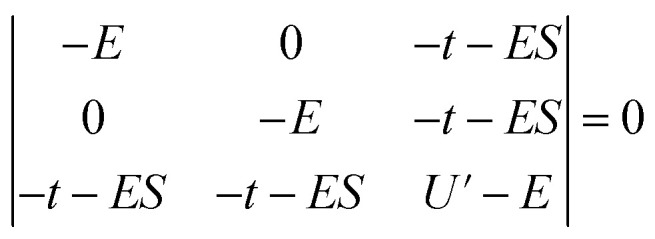


The resulting energies are 0 and 
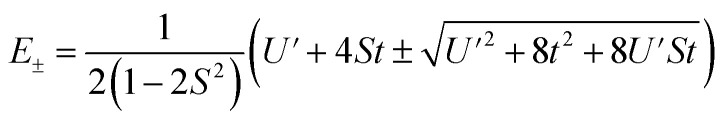
. The wavefunctions of interest are *ψ*_±_ = *N*(|L^1^ 4f^1^〉 + *λ*|L^2^ 4f^0^〉) where 
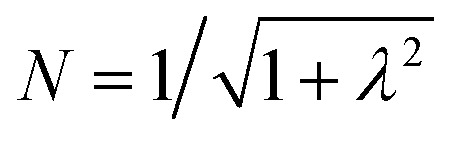
, and 
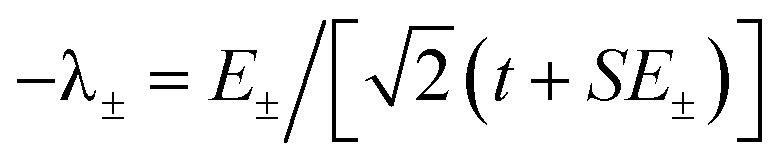
. In addition, 
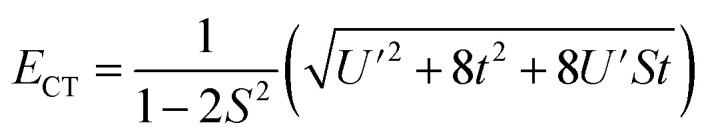
 and *n*_f_ = *N*^2^. Because there are three parameters, *t*, *S*, and *U*′, their values cannot be determined from *E*_CT_ and *n*_f_. To determine *E*_−_ to second order using the HMM, two approximation must be made. The first is that *t* can be estimated using the Wolfsberg–Helmholz (WH) approximation, −*t* = *S*[*E*(|L^1^ 4f^1^〉) + *E*(|L^2^ 4f^0^〉)], where *E*(|L^1^ 4f^1^〉) and *E*(|L^2^ 4f^0^〉), are the energies of the |L^1^ 4f^1^〉 and |L^2^ 4f^0^〉 basis states, respectively.^38^ This form of the WH approximation is slightly different from that originally proposed by Wolfsberg and Helmholz, *H*_*ij*_ = (*KS*)(*H*_*ii*_ + *H*_*jj*_)/2, where *K* is a constant equal to 1.67 for σ interactions and 2.00 for π interactions.^38^ The difference is that the simplified form assumes *K* = 2 for both σ and π interactions, which makes no difference for fitting. However, if one wanted to determine the atomic orbital overlap integrals, the group overlap, *S*, would have to be adjusted accordingly.

The other approximation is that the energy of the highest occupied VB basis state in all LnO_2_ is the Fermi level (*E*_F_) of CeO_2_, −7.4 eV, which is the valence band maximum for the O 2p band of CeO_2_.^56^ This energy corresponds to those states with the least stabilization due to mixing between the O 2p and Ce 4f/5d orbitals and includes electrostatic effects, which makes it an appropriate approximation for the energy of the VB basis states.

Using these approximations, *t* can be written in terms of *E*_F_, *U*′, and *S*, *t* = –*S*[*E*_F_ + (*E*_F_ + *U*′)] with *E*_F_ = −7.4 eV, and the HMM can be solved to second order using *S* and *U*′ as the only parameters. The results are given in Table 4.

**Table 4:** Stabilization of LnO_2_ to second order. Group overlap, *S*, is unit-less, and energies are in eV

	CeO_2_	PrO_2_	TbO_2_
*S* 4f	0.102(3)	0.067(4)	0.059(5)
*U*′ 4f	0.4(3)	0.94(9)	2.4(1)
*t* 4f	1.46(5)	0.93(5)	0.74(5)
*E*_−_ 4f	−1.6(2)	−0.85(7)	−0.37(5)
Total 4f stabilization	−1.6(2)	−0.85(7)	−0.74(9)
*S* 5d(e_g_)	0.164(3)	0.165(3)	0.157(3)
*U*′ 5d(e_g_)	2.3(4)	2.0(3)	2.2(3)
*t* 5d(e_g_)	2.06(8)	2.10(7)	1.98(7)
*E*_−_ 5d(e_g_)	−1.6(2)	−1.7(2)	−1.6(2)
Total 5d(e_g_) stabilization	−3.3(3)	−3.5(3)	−3.2(3)
*S* 5d(t_2g_)	0.239(3)	0.238(3)	0.231(3)
*U*′ 5d(t_2g_)	2.2(6)	1.8(7)	2.1(6)
*t* 5d(t_2g_)	3.0(1)	3.1(1)	2.9(1)
*E*_−_ 5d(t_2g_)	−2.5(3)	−2.7(3)	−2.5(3)
Total 5d(t_2g_) stabilization	−7.5(8)	−8(1)	−7.4(8)

### Stabilization of CeO_2_ by 4f orbital interactions determined from magnetic susceptibility

To check the accuracy of the stabilization calculated using the HMM, it would be helpful to determine this energy independently. The stabilization of the ground state of CeO_2_ by 4f orbital interactions can potentially be determined from its temperature-independent susceptibility (*χ*_TIP_) as previously done for [C_8_H_8_]_2_Ce and 
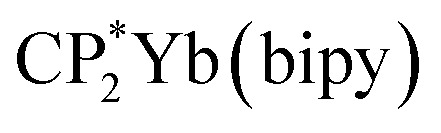
, where Cp* is pentamethylcyclopentadienyl and bipy is 2,2′-bipyridyl.^36,37^ The stabilization of *Ψ*_−_ (the singlet ground state) in Fig. 2 can be modeled as antiferromagnetic coupling between an unpaired electron in the Ce 4f orbitals and an unpaired electron in the O 2p orbitals.^57^ The Heisenberg–Dirac–van Vleck spin Hamiltonian for this interaction is given by eqn (3) where 2*J* is the stabilization of the singlet state relative to the triplet state, and *S*_O˙−_ and *S*_Ce(iii)_ are the spins of the electrons in the O_2p_ and Ce 4f orbitals, respectively. As shown by Griffith using eqn (4), *χ*_TIP_ of the singlet state is related to the difference in energy between the triplet and singlet states and the spectroscopic splitting factors (*g*-values) of the electrons, and the other symbols have their usual meanings.^57^*χ*_TIP_ is due to the orbital angular momentum of the electrons in the ground state, *Ψ*_−_ = *N*(|L^1^ 4f^1^〉 + *λ*|L^2^ 4f^0^〉); however, only the |L^1^ 4f^1^〉 component contributes to the TIP. Therefore, eqn (4) must be modified to reflect this fact as given in eqn (5), which takes into account that *N*^2^ is equal to *n*_f_ in this case.^58^ The *g* values for a O˙^−^ radical have been measured in ice and are (2.0632, 2.0829, and 2.0027).^59^ The *Γ*_7_ basis state of Ce(iii) consists of states derived from the *J* = 5/2 ground state manifold and the *J* = 7/2 excited state manifold, *Γ*_7_ = *a*|5/2, *Γ*_7_〉 + *b*|7/2, *Γ*_7_〉, where *a*^2^ + *b*^2^ = 1 and *a*^2^ should be close to unity.^60^ The *g*-value of *Γ*_7_ is given by eqn (6).3
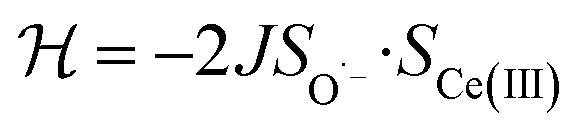
4
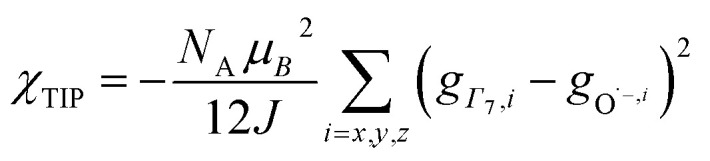
5
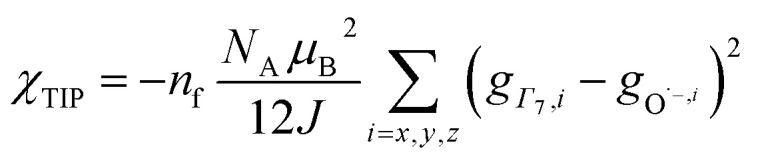
6
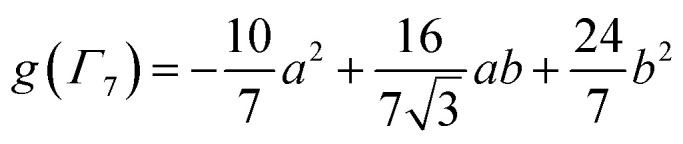


The measured values *χ*_TIP_ for Ce^4+^ in CeO_2_, corrected for inherent diamagnetism, vary from 15 × 10^−6^ emu mol^−1^ to 54 × 10^−6^ emu mol^−1^.^61–66^ We measured the magnetic susceptibility of two, high purity commercial samples, which had been dried under vacuum for 2 days to remove adsorbed water (Fig. S7 and S8†). The samples yielded similar values for *χ*_TIP_, 70.2 × 10^−6^ emu mol^−1^ and 72.0 × 10^−6^ emu mol^−1^, with an average of 71.1(9) ×10^−6^ emu mol^−1^. Assuming that *Γ*_7_ consists entirely of the |5/2, *Γ*_7_〉 state (*a*^2^ = 1 in eqn (6)) with *g*(*Γ*_7_) = −10/7, eqn (5) yields a value of −1.5(1) eV for 2*J*, which is consistent with the values of *E*_*−*_ determined using the HMM, −1.9(2) eV and −1.6(2) eV to first and second order, respectively. Although determination of 2*J* is not completely independent from *E*_−_ (both include *n*_f_), the agreement supports the validity of the HMM for determining the stabilization due to a specific orbital interaction.

## Discussion

There are two main areas for discussion: use of the HMM to determine stabilization due to specific orbital interactions, and examining those values in the lanthanide dioxides. Our interest in the HMM is primarily to determine how much stabilization a given orbital interaction provides in a molecule, a complex, or an extended solid. As shown by Hubbard *et al.*, to first order this energy is given by the amount of excited state character mixed into the ground state multiplied by the energy of the corresponding CT band.^34^ Both of these values may be determined from a single O K-edge XANES spectrum under ideal conditions since the area of the pre-edge peaks can be used to determine the degree of mixing, and *E*_CT_ can be determined by fitting pre-edge peaks if the offset between the energies of the CT bands and of the O K-edge XANES pre-edge peaks are known. In this study, that value is 525.9(1) eV. While this value might be transferrable to other, closely related systems, *e.g.*, the actinide dioxides, it is unlikely that it can be directly applied to other systems. Minasian *et al.* also found a linear relationship between the XANES pre-edge peaks and the CT bands of MO_4_^2−^ with a similar energy offset, 526.9(1) eV, but a different slope, 0.81(2).^50^ For other systems, the offset between *E*_BG_ and *E*_PE-BG_ can be determined using the approach described here: measuring *E*_BG_ and comparing it to *E*_PE-BG_. As noted above, the relationship between the XANES pre-edge peaks and the CT bands is well known.^50,52^

Our main reason for using the HMM was to estimate how much stabilization the interactions between ligand orbitals and metal 4f and 5d orbitals provide, so the parameter of most interest to us is the total stabilization in Tables 3 and 4. The values of the other parameters can be used to examine whether the HMM is internally coherent and consistent with the trends observed for the 4f and 5d orbitals among Ce, Pr, and Tb. Since the HMM uses a basis set that does not include orbital interactions between the ligand and metal orbitals, the value of *U*′ for the 5d(e_g_) interaction should be identical that of the 5d(t_2g_) interaction. As shown in Tables 3 and 4 these values of *U*′ are the same given their uncertainties.

For both CeO_2_ and PrO_2_, the basis VB state |L^1^ 4f^1^〉 is lower in energy than |L^2^ 4f^0^〉 because *n*_f_ > 0.5. The ground |L^1^ 4f^1^〉 state used in the HMM may seem to imply that CeO_2_ has a localized 4f electron in CeO_2_; however, this is not the case. The HMM result suggests that the *Ψ*_−_ state is a delocalized, bonding state as indicated by the large degree of mixing of the excited |L^2^ 4f^0^〉 state into the ground state. The HMM result for CeO_2_ is equivalent to an MO description with the doubly-occupied O 2p SALC with A_2u_ symmetry possessing just over 25% Ce 4f character. The HMM result for CeO_2_ is also consistent with the interatomic intermediate-valence description, which reaches the same conclusion from an Anderson impurity model.^10^

A different approach to correct the HMM to second order was described by Hubbard *et al.*^34^ The one used here takes advantage of the fact that the Hubbard hopping integral, *t*, is equivalent to the interaction energy in MO theory, which can be modeled using the Wolfsberg–Helmholz approximation. The HMM can be used to estimate the stabilization energies if additional data is available. Here, the energy of the higher lying basis state is approximated using the valence band maximum of CeO_2_. As expected, the stabilization due to orbital interactions is smaller in the second-order model than the first order model. For CeO_2_ the stabilization determined to second order, −1.6(2) eV, is in good agreement with the value determined from the TIP of CeO_2_, −1.5(1) eV. An alternative approach is to compare the results from the HMM with those from computation. The electronic structures of LnO_2_, especially that of CeO_2_, have been extensively studied including the amount of orbital mixing (*n*_f_) and the relationship between *n*_f_ and the XANES spectra.^46,67–72^ Most studies of LnO_2_ focus on spectroscopic or other physical properties rather than bonding. However, the value of *t* may be compared to the value of *V*_kf_ determined from modeling photoemission spectra. For CeO_2_, the value of *V*_kf_ was found to be between 1.1 eV and 1.8 eV, which is consistent with the values of *t*, 1.51(5) and 1.46(5) eV, in the first and second order models, respectively.^73^

Use of the HMM allows the contributions of the 4f and 5d orbitals to bonding in LnO_2_ to be examined as one moves across the lanthanide series. The contributions of the 4f orbitals to bonding vary as one moves from CeO_2_ to TbO_2_ as given in Table 4. The 4f orbitals provide the greatest stabilization in CeO_2_, 1.6(2) eV, and less in PrO_2_ and TbO_2_, 0.85(7) and 0.74(9) eV, respectively. The origin of this trend is the change in the group overlap integral, *S*, which decreases from 0.102(3) in CeO_2_ to 0.067(4) and 0.059(5) in PrO_2_ and TbO_2_, respectively. Qualitatively, this behavior is expected since the 4f orbitals have their greatest radial extent at the beginning of the lanthanide series and contract due to increasing effective nuclear charge as one proceeds to higher atomic numbers.

Unlike the 4f orbitals, the 5d orbitals vary little in their contribution to the stabilization of LnO_2_ as shown by the results in Table 4. This consistency was also observed in the 5d interactions in octahedral, trivalent LnCl_6_^3−^ complexes studied by Jung *et al.*^74^ In LnO_2_, the stabilization provided by the 5d e_g_ orbitals is 3.2(3) to 3.5(5) eV, and that provided by the 5d t_2g_ orbitals is 7.4(8) to 8(1) eV. The lack of variation reflects the similar values for the group overlaps of the O 2p orbitals with the 5d e_g_ orbitals, 0.157(3) to 0.165(3) and with the 5d t_2g_ orbitals, 0.231(3) to 0.239(3). The interactions between the O 2p orbitals and the Ln 5d orbitals stabilize the compounds by 11(1), 12(1), and 11(1) eV in CeO_2_, PrO_2_, and TbO_2_, respectively. In comparison, the stabilization provided by the 4f interactions is roughly an order of magnitude smaller, 1.6(2) eV (37 kcal mol^−1^) in CeO_2,_ and less in PrO_2_ and TbO_2_. This large difference in stabilization was also seen in the calculations of Li *et al.*^32^ The smaller role played by the 4f orbitals is largely consistent with the FEUDAL model for bonding in the lanthanides, which suggests that the ligand electrons are primarily stabilized by interaction with the 5d rather than the 4f orbitals.^8^

For comparison with other studies of bonding in Ln complexes, the energies of these interactions may be examined using angular overlap parameters for cubic coordination. For the 4f_*xyx*_ orbital, the energy is (40/9)*e*_σ,4f_.^75^ For the 5d orbitals, the e_g_ orbital energy is (16/3)*e*_π,5d_ and the t_2g_ orbital energy is (8/3)*e*_σ–5d_ + (18/8) *e*_π–5d_.^76^ Using these relationships, the values of *e*_σ_ and *e*_π_ for the 4f and 5d interactions in LnO_2_ can be determined and are given in Table 5.

**Table 5:** Angular overlap model energies for LnO_2_

	*e*_σ,4f_ (cm^−1^)	*e*_σ,5d_ (cm^−1^)	*e*_π–5d_ (cm^−1^)
CeO_2_	2900	18 000	5000
PrO_2_	1600	20 000	5300
TbO_2_	1300	19 000	4800

Despite their reduced role relative to the 5d orbitals, the 4f orbitals do stabilize these complexes as illustrated by the 1.6(2) eV stabilization provided in CeO_2_. The degree of stabilization provided by the 4f orbitals in CeO_2_ is greater, likely much greater, than expected for Ln compounds other than LnO_2_ for two main reasons. The overlap between the Ln 4f_*xyz*_ orbital and the O 2p ligands is expected to be larger relative to Ln complexes other than LnO_2_ because the lobes of the 4f_*xyz*_ orbital point directly at the eight oxide ligands (all interactions are σ-interactions) and because Ce is at the beginning of the lanthanide series, so the 4f orbitals are less contracted relative to those of the later Lns. The second reason is that the difference in energy between the Ce 4f orbitals and O 2p orbitals is smaller relative to a formally trivalent Ln complex because 4f orbitals are lower in energy in a formally tetravalent Ln complex. Both of these factors increase orbital mixing and the strength of the Ce 4f/O 2p interaction relative to trivalent Ln complexes.

The contributions of the 4f and 5d orbitals to bonding may be compared with the stabilization provided by electrostatic effects.^21^ Interactions between the O 2p orbitals and the Ln 4f and 5d orbitals reported in Table 4 provide approximately the same amount of stabilization: 12(1), 12(1) and 11(1) eV for CeO_2,_ PrO_2_ and TbO_2_, respectively. The lattice energies (*U*) and Madelung energies of LnO_2_ were determined by Angelow using a Born–Haber cycle (Table 6).^21^ The lattice energy includes both electrostatic effects and orbital interactions, and the electrostatic stabilization of LnO_2_ increases as the atomic number of Ln increases due to the decreasing ionic radii of Ln^3+^ with increasing atomic number. We calculated the electrostatic contribution, *E*_BL_, using the Born–Landé equation (eqn (7)), where *N*_A_ is Avogadro's number, *Z*^+^ and Z^+^ are the charges on the positive and negative ions, respectively, *M* is the Madelung constant, *e* is the charge of the electron, *ε*_0_ is permittivity of free space, *r* is the distance between the cation and anion, and *n* is the Born exponent with *M* = 2.51939, the value for the CaF_2_ lattice. The values of the Born exponent, *n*, were determined from the derivative of the bulk modulus, *B*, with respect to pressure, d*B*/dP, using eqn (8), where *n* is the Born exponent.^77^ Measured values of d*B*/d*P* for CeO_2_ and PrO_2_ were reported by Gerward *et al.*, and d*B*/d*P* for TbO_2_ was determined from the pressure dependence calculated by Miran *et al.*^78,79^ As is clear from the values of *E*_BL_, the stabilization due to electrostatic effects is much larger than the stabilization due to orbital overlap; as in trivalent lanthanide compounds, bonding in LnO_2_ is best described as ionic with a small contribution from orbital interactions (covalent bonding). The rationale for determining *E*_BL_ was to determine whether the trends in 4f and 5d bonding among the LnO_2_ were reflected in lattice energy once the effect of ionic bonding had been accounted for. Due to the uncertainty in *E*_BL_, the only conclusion we are able to draw is that *U* − *E*_BL_ is approximately equal for LnO_2_ which is consistent with the trend in stabilization provided by interactions between the O 2p orbitals and Ln 4f and 5d orbitals (Table 5).7
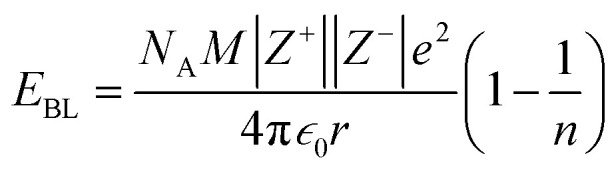
8
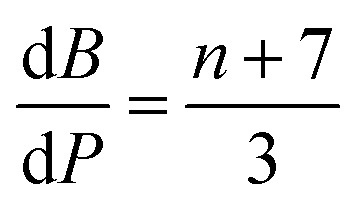


**Table 6:** Lattice energy and electrostatic stabilization of LnO_2_^a^

	*a* (Å)	d*B*/d*P*^78,79^	*n*	*U* (eV)^21^	*E*_BL_ (eV)	*U* − *E*_BL_ (eV)
CeO_2_	5.411	4.4(4)	6.2(1.2)	109.4(3)	104(4)	6(4)
PrO_2_	5.393	4.8(5)	7.4(1.5)	110.7(3)	107(3)	3(3)
TbO_2_	5.213	4.5(5)^b^	6.5(1.5)	113.2(3)	109(5)	5(5)

a *a* is the lattice parameter, d*B*/d*P* is the is the derivative of bulk modulus with respect to pressure, *n* is the Born exponent, *U* is the lattice energy determined from the Born–Haber cycle, *E*_BL_ is the is electrostatic stabilization of the lattice calculated using the Born–Lande equation.

b Relative uncertainty assumed to be 10% based on the measured values for CeO_2_ and PrO_2_.

A final area for discussion is the relationship of the HMM to related electronic structure models that include CT interactions. The earliest such model that we are aware of is the one proposed by Hubbard, which was described in greater detail above.^34^ Fox and Matson developed a Hückel plus CI model for π-bonding in ethylene.^80^ The valence bond configuration interaction (VBCI) model, developed by Kennepohl and Solomon for use with photoelectron measurements, has been used for investigating bonding in first row transition metal complexes.^81^ The main use of the VBCI model has been to determine the degree of covalency in metal ligand bonds.^82^ Since these models were derived using perturbation theory to mix CT states into the ground state, they are all similar mathematically. They differ superficially in the nature of the parameters in the models. For example, *U*′ in the HMM is equivalent to *Δ*–*Q* in the VBCI. Where they differ substantially is in their application. The Hückel plus CI model is aimed at π-electron systems. The HMM and VBCI are similar models with different applications. While both models focus on bonding, the primary use of VBCI has been to examine the degree of covalency in metal–ligand bonds, whereas the primary use of the HMM is to determine how much stabilization is provided by covalent bonding. In addition to these relatively simple models, more complex models including CTM4XAS^83^ and the previously mentioned Anderson impurity model,^13,14,73^ include mixing of CT states into the ground state. As with the simpler models, the more complex models have different goals than HMM. CTM4XAS is a comprehensive package for determining the nature of the ground state, including the degree of covalency in metal–ligand bonds, by simulating XAS spectra. The Anderson impurity model has been primarily used for obtaining similar information by modeling photoelectron spectra as well XAS spectra. The HMM is complementary to previously reported models in that the input for the HMM is the degree of covalency, which is the product of most of the related models.

## Limitations of this study

As used here, the HMM has a number of limitations. The primary issue is that the HMM is a molecular model and LnO_2_ are extended solid state compounds not molecules. Applying the HMM to LnO_2_ makes the implicit assumption that the bonding in LnO_2_ can be represented by a single LnO_8_^12−^ cluster. A more appropriate model for LnO_2_ is the Anderson impurity model, which captures more of the interactions among the electrons of LnO_2_ and allows one to model other spectroscopic properties.^13,14,73^ The more complete Anderson impurity model for LnO_2_ requires five variables, which cannot be fit using only *n*_f_ and *E*_CT_. Our main goal is to determine the contribution of 4f and 5d orbital interactions to the stabilization of LnO_2_, so use of the HMM seems appropriate. The agreement between the stabilization of CeO_2_ due to 4f orbital interactions determined using the HMM and from the TIP of CeO_2_ suggests that the assumptions implicit in using the HMM are valid.

The other main limitation of the HMM is that it is a semiempirical model that relies on the accurate measurement of UV-vis and X-ray absorption spectra and correct assignment of their features. While the assignment of the lowest energy CT band is often straightforward, this is not always true, and assignment of higher energy CT bands is challenging. In lanthanide compounds, it is difficult to differentiate between CT bands and 4f to 5d transitions, which are both allowed. In some cases, assignment may be simplified by comparing the features in the UV-vis spectra to the pre-edge features in the ligand K-edge XANES spectra since the pre-edge peaks only correspond to charge-transfer peaks.^50^ However, the low energy, “soft” X-rays required to probe the K-edge for light elements such as oxygen (*ca.* 530 eV) present technical challenges.^20,50,84–88^ Techniques for the accurate measurement of ligand K-edge spectra are described elsewhere, along with appropriate methods for data reduction and quantification of transition energies and intensities.^20,50,84–88^ Even for high-symmetry systems, assignment of ligand K-edge XANES spectra is greatly aided by calculation.^89–93^ For example, assignments for the pre-edge features in the O K-edge XANES spectra of LnO_2_, in which the peaks are well-resolved, were validated by comparison with results from density-functional theory calculations.^18^

There are two issues with the values of *n*_f_ and *n*_d_ in this study. The value of *n*_f_ has a small error due the fact that CI with the |L^0^4f^2^〉, “Ce(ii) like” state is not included in the data analysis. This error is estimated to be less than 5%.^46^ The other issue is that *n*_d_(t_2g_) may be slightly inflated relative to its “true” value due to inclusion of Rydberg transitions. The O K-edge XANES pre-edge peak associated with 5d(t_2g_) is broad and close to the O K-edge itself, and weak transitions to Rydberg states occur at approximately the same energy.^86^ Contributions from the Rydberg transitions increase the areas of the peaks assigned to 5d(t_2g_) final states, which in turn inflate the values of *n*_d_(t_2g_).

The final issue is that the first-order HMM overestimates the stabilization due to orbital interactions. If the overlap is small, the error is small as can be seen by comparing stabilization by the 4f orbitals in Tables 3 (first order) and 4 (second order). For this reason, the first order HMM is likely to be most appropriate for lanthanide and actinide systems due to the small overlap between ligand orbitals and 4f and 5f orbitals. The first-order HMM is less accurate when the overlap is larger.

Despite these limitations, the HMM provides electronic structure information that would otherwise be challenging to obtain experimentally. Specifically, the HMM provides the stabilization due to a specific orbital interaction. In other words, the HMM allows one to experimentally determine the contribution of covalent interactions to bond strength analogously to the manner in which the Madelung energy allows one to estimate the electrostatic contribution to bond strength. Use of the HMM in this way relies on XANES spectroscopy, especially ligand K-edge XANES spectroscopy, to determine the amount of excited state character (*n*_f_ and *n*_d_) mixed into the ground state. Determining the *n*_f_ for lanthanide complexes can be straightforward if the information is available from the Ln L_3_-edge as originally proposed by Dexpert *et al.*^9^ Ligand K-edge spectra are complementary in that they can provide both *n*_f_ (or *n*_d_) and *E*_CT_ under ideal circumstances.

## Conclusion

The stabilization of LnO_2_ by interactions between the O 2p and Ln 4f and 5d orbitals was determined experimentally with a HMM using the amount of excited charge-transfer state character mixed into the ground state and the energy of charge-transfer state. The amount of the charge-transfer state character mixed into the ground state was determined by a combination of Ln L_3_-edge XANES and O K-edge XANES spectroscopy. The energies of the charge-transfer states were determined from the energies of the pre-edge peaks in the O K-edge XANES spectrum by determining the difference between the energies of the band gaps in diffuse reflectance and the band gaps for the O K-edge XANES pre-edge peaks, 525.9(1) eV.

The stabilization due to orbital interactions (covalent contribution to the bond strength) was determined to first and second-order. The contribution of the 4f orbitals to bonding in CeO_2_ was 1.6(2) eV and was smaller in PrO_2_ and TbO_2_. The combined contributions from the 5d orbitals was approximately 11 eV in all compounds. The ionic contribution to bonding was determined using the Born–Landé formula and was 104(4) eV in CeO_2_ to 109(5) eV in TbO_2_. As expected, bonding in these compounds is overwhelmingly ionic with a minor covalent contribution from interactions between the O 2p and Ln 4f and 5d orbitals. Within the covalent contribution to bonding, the FEUDAL model is largely correct. The contribution from the 4f orbitals is ∼10% of that from the 5d orbitals. The stabilization provided covalent bonding *via* the 5d orbitals is in turn dwarfed by the electrostatic stabilization of LnO_2_.

More generally, this study shows how covalent contributions to bonding may be determined spectroscopically. The spectroscopy used here, O K-edge XANES, can be applied to other metal oxide systems or covalent molecules such as carbon monoxide. Once the relative energy scales of the CT bands and O K-edge pre-edge peaks are determined, the CT energies as well as the orbital mixing can be determined from the O K-edge XANES spectra. The stabilization due to these interactions can then be determined using the HMM. As quantitative mixing information is available from the K-edge XANES spectra of additional chemical elements, this approach can be expanded to new systems.

## Data availability

DR spectra of LnO_2_, shown in Fig. 3, are available from the authors.

## Author contributions

Lukens made the compounds, performed the diffuse reflectance and magnetic susceptibility measurements, and developed the HMM for LnO_2_. Booth and Minasian contributed the analyses of the Ln L_3_-edge spectra and O K-edge spectra, respectively, especially the relationship between the areas of the peaks and *n*_f_ and *n*_d_. All authors determined the relationships between the HMM and spectroscopic data. All authors contributed to the manuscript.

## Conflicts of interest

There are no conflicts to declare.

## Supplementary Material

SC-014-D3SC03304J-s001
