# Supplementary material for: Strengths of covalent bonds in LnO2 determined from O K-edge XANES spectra using a Hubbard model
Source: Chem Sci. 2023 Nov 3;14(44):12784–95. doi: 10.1039/d3sc03304j (PMC10646950; doi:10.1039/d3sc03304j)
Supplement: SC-014-D3SC03304J-s001 [file SC-014-D3SC03304J-s001.pdf]

Electronic Supplementary Material (ESI) for Chemical Science.
This journal is © The Royal Society of Chemistry 2023

Supplementary Information for

Strengths of covalent bonds in LnO_2 determined from O K-edge XANES spectra using a Hubbard model

Wayne W. Lukens*, Stefan G. Minasian, and Corwin H. Booth

Chemical Sciences Division
Lawrence Berkeley National Laboratory
Berkeley, CA 94720

Contents

Hubbard molecule model for CeO_2: O 2p and Ce 4f, A_{2u} mixing	S2
Hubbard molecule model for PrO_2: O 2p and Ln 4f, A_{2u} mixing	S3
Hubbard molecule model for TbO_2: O 2p and Ln 4f, A_{2u} mixing	S4
Hubbard molecule model for LnO_2: O 2p and Ln 5d, E_g mixing	S5
Hubbard molecule model for LnO_2: O 2p and Ln 5d, T_{2g} mixing	S6
HMM to second order for CeO_2 4f interaction	S7
HMM to second order for PrO_2 and TbO_2 4f interactions.	S8
HMM to second order for LnO_2: O 2p and Ln 5d interactions	S9
Experimental details	S10
References	S11
**Figure S3**. Tauc plots for CeO_2 from the data published by Niwano, et al.	S12
**Figure S4**. Tauc plots for CeO_2 O K-edge XANES pre-edge peaks.	S13
**Figure S5**. Tauc plots for PrO_2 O K-edge XANES pre-edge peaks.	S14
**Figure S6**. Tauc plots for TbO_2 O K-edge XANES pre-edge peaks.	S15
**Figure S7.** Magnetic Susceptibility of CeO_2 from Aldrich.	S16
**Figure S8.** Magnetic Susceptibility of CeO_2 from Strem.	S16

Hubbard molecule model for CeO_2: O 2p and Ce 4f, (A_{2u}) mixing

For CeO_2, the unperturbed ground state is $|L^1 4f^1\rangle$, which consists of four wavefunctions ψ_1-ψ_4. This state corresponds to a "hole" in the O 2p A_{2u}^1 orbital (L^1) and one electron in the Ce 4f A_{2u} orbital ($4f^1$), which is the main component of the Γ_7 state if spin orbit coupling is included. The energy of ψ_1-ψ_4 is assigned as 0. The excited state that interacts with $|L^1 4f^1\rangle$ is $|L^2 4f^0\rangle$ and consists of a single wavefunction ψ_5. The energy of $|L^1 4f^1\rangle$ is set to zero, and the energy of $|L^2 4f^0\rangle$ is U'. The individual states are shown in Table S1. Only states in which the electron can hop from the occupied O 2p A_{2u} orbitals into the Ce 4f orbital without changing sign may interact with ψ_5; these states are ψ_1 and ψ_4. In other words, only ψ_1 and ψ_4 have non-zero, off-diagonal matrix elements. The other states cannot interact with ψ_5 and are excluded from the model.

Table S1: A_{2u} symmetry, L^1 $4f^1$ basis states for the Hubbard molecule model of LnO_2. Only the states in red are used in the model.

	$4f^1$ (↑)	$4f^1$ (↓)
L^1 (↓)	ψ_1	ψ_3
L^1 (↑)	ψ_2	ψ_4

ψ_5 is $|L^2\Gamma_7{}^0\rangle$

The Hubbard molecule model Hamiltonian for the interaction between the $|L^1 4f^1\rangle$ and $|L^2\Gamma_7{}^0\rangle$ in CeO_2 can be described using the following matrix,

$$\boldsymbol{A} = \begin{pmatrix} 0 & 0 & -t \\ 0 & 0 & -t \\ -t & -t & U' \end{pmatrix}, \text{ where } \begin{pmatrix} 1 \\ 0 \\ 0 \end{pmatrix} = \psi_1, \begin{pmatrix} 0 \\ 1 \\ 0 \end{pmatrix} = \psi_4, \text{ and } \begin{pmatrix} 0 \\ 0 \\ 1 \end{pmatrix} = \psi_5,$$

t is the electron hopping term (interaction integral), which is analogous to the off-diagonal matrix element, H_{ij}, (orbital interaction integral) in MO theory, and U is the energy of the unperturbed excited state with respect to the ground state.

The eigenvalues are 0 and $E_\pm = \frac{1}{2}\left(U' \pm \sqrt{U'^2 + 8t^2}\right)$. The eigenvectors of interest are $\psi_\pm$, which have energies $E_\pm$. The eigenvectors are determined from $\mathbf{A}$-$E_\pm\mathbf{I} = \mathbf{0}$. From the first 2 rows of $\mathbf{A}$, $a_1 = a_4$ and $E_\pm a_1$=-ta_5 or $a_5 = -(E_\pm/t)\,a_1$, where a_i is the coefficient of ψ_i. Using $a_1 = 1$, the unnormalized wasvefunction is $\psi_\pm = \psi_1 + \psi_4 - (E_\pm/t)\psi_5$. The normalization constant, N, is $1/\sqrt{2 + (E_\pm/t)^2}$. The $\psi_\pm$ can be simplified by dividing by $\sqrt{2}$ to give $\psi_\pm = N\left[(\psi_1 + \psi_4)/\sqrt{2} - \left(E_\pm/\sqrt{2}\,t\right)\psi_5\right]$, with N=$1/\sqrt{1 + \left(E_\pm/\sqrt{2}t\right)^2}$. Finally, $(\psi_1 + \psi_4)/\sqrt{2}$ can be more conveniently represented as $|L^1 4f^1\rangle$ and ψ_5 by $|L^2 4f^0\rangle$, to give the following:

$E_\pm = \frac{1}{2}\left(U' \pm \sqrt{U'^2 + 8t^2}\right)$ and $\psi_\pm = N(|L^1\ 4f^1\rangle + \lambda|L^2\ 4f^0\rangle)$ where N=$1/\sqrt{1 + \lambda^2}$, $\lambda = -E_\pm/\sqrt{2}t$.

$E_{MLCT} = \sqrt{U'^2 + 8t^2}$ and $n_f = 1/N^2$.

Hubbard molecule model for PrO_2: O 2p and Ln 4f, A_{2u} mixing

The ground state of Pr^{4+} in cubic symmetry is $4f^1$. As discussed for CeO_2, the f-orbital that most strongly interacts with the oxygen orbitals is A_{2u}, which is unoccupied in Pr^{4+}. Since the only portion of the Pr wavefunction involved in the HMM is the single electron in the A_{2u} orbital, the HMM for PrO_2 is identical to that of CeO_2.

Hubbard molecule model for TbO_2: O 2p and Ln 4f, A_{2u} mixing

Unlike CeO_2 and PrO_2, the unperturbed ground state of TbO_2 in the HMM involves a tetravalent lanthanide and has two electrons in the OA_{2u} (L^2) orbital. In the unperturbed ground state, $|L^2 4f^7\rangle$, the oxygen A_{2u} orbital is doubly occupied and each of the f-orbitals is singly occupied. While the actual ground state is an octet, in the HMM, the ground state is simplified to a doublet: ψ_1 is spin up and ψ_2 is spin down. Likewise, in the unperturbed charge transfer state, the oxygen A_{2u} orbital is singly occupied, and the corresponding orbital on Tb is double occupied: ψ_3 is spin up and ψ_4 is spin down. Only ψ_3 can interact with ψ_1, and only ψ_4 can interact with ψ_2. The energy of $|L^2 4f^7\rangle$ is set to zero, and the energy of $|L^1 4f^8\rangle$ is U'.

Table S1: A_{2u} symmetry, L^1 $4f^8$ basis states for the Hubbard molecule model of TbO_2.

	$4f^8$ (↓↑)
L^1 (↑)	ψ_3
L^1 (↓)	ψ_4

ψ_1 is $|L^2 4f^7\uparrow\rangle$; ψ_2 is $|L^2 4f^7\downarrow\rangle$

The Hubbard molecule model Hamiltonian for TbO_2 can be described using the following matrix, **A**,

$$\boldsymbol{A} = \begin{pmatrix} 0 & 0 & -t & 0 \\ 0 & 0 & 0 & -t \\ -t & 0 & U' & 0 \\ 0 & -t & 0 & U' \end{pmatrix}, \text{ where } \begin{pmatrix} 1 \\ 0 \\ 0 \\ 0 \end{pmatrix} = \psi_1, \begin{pmatrix} 0 \\ 1 \\ 0 \\ 0 \end{pmatrix} = \psi_2, \begin{pmatrix} 0 \\ 0 \\ 1 \\ 0 \end{pmatrix} = \psi_3, \text{ and } \begin{pmatrix} 0 \\ 0 \\ 0 \\ 1 \end{pmatrix} = \psi_4$$

The eigenvalues for the model are $E_\pm = \frac{1}{2}\left(U' \pm \sqrt{U'^2 + 4t^2}\right)$. The only eigenvectors we care about are $\psi_\pm$, which have energies $E_\pm$. The eigenvectors are determined by solving $\mathbf{A}$-$E_\pm\mathbf{I} = \mathbf{0}$. From the first 2 rows of **A**, we get $E_\pm a_2$=-t a_4 and $E_\pm a_1$=-t a_3, where a_i is the coefficient of ψ_i. The ground and excited states are doubly degenerate. Using $a_1 = 1$, we get $\psi_{\pm 1} = \psi_1 - (E_\pm/t)\psi_3$ and $\psi_{\pm 2} = \psi_2 - (E_\pm/t)\psi_4$, which is not normalized. The normalization constant, N, is $1/\sqrt{1 + (E_\pm/t)^2}$. For TbO_2, the HMM gives the following result:

$E_\pm = \frac{1}{2}\left(U' \pm \sqrt{' + 4t^2}\right)$, $\psi_{\pm 1}$ = N[$|L^2 4f^7\uparrow\rangle + \lambda|L\uparrow 4f^8\rangle$]; $\psi_{\pm 2}$ = N[$|L^2 4f^7\downarrow\rangle + \lambda|L\downarrow 4f^8\rangle$] where N=$1/\sqrt{1 + \lambda^2}$ and $\lambda = -E_\pm/t$.

$E_{MLCT} = \sqrt{U'^2 + 4t^2}$ and $n_f = 2(\lambda^2/N^2)$.

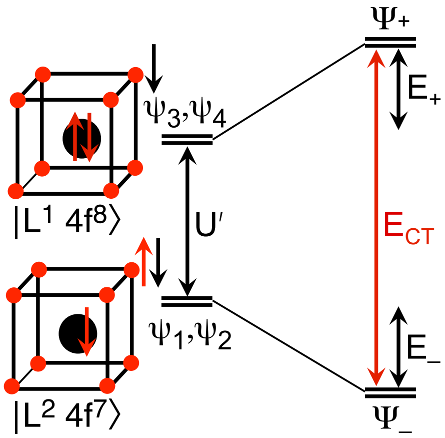

Figure S1. HMM for the 4f interaction in TbO_2.

Hubbard molecule model for LnO_2: O 2p and Ln 5d, E_g mixing

The ground state is $|L^4E_g^0\rangle$, which corresponds to O 2p E_g^4 and Ln 5d E_g^0. This state is spatially degenerate, and the model may be more conveniently applied by considering each component of the ground state separately since they interact with different excited states. The spatially degenerate wavefunctions of ground state are given by ψ_1 and ψ_2, which correspond to the empty d_{z^2} and empty $d_{x^2-y^2}$ orbitals and the matching, filled O 2p SALCs, respectively. The energy of ψ_1 and ψ_2 is 0. The excited state that interacts with ψ_1 is $|L^3E_g^1\rangle$, which 16-fold degenerate and given by ψ_3 - ψ_{18}. The individual states are shown in Table S1. Only states in which the electron can hop from the occupied O 2p E_g orbitals into the Ln 5d E_g orbitals without changing sign can interact with the ground state: ψ_{1a} can interact with ψ_2 and ψ_7, and ψ_{1b} can interact with ψ_{12} and ψ_{17}. The other states cannot interact with ψ_1 and are excluded from the model.

Table S1: E_g^3 5d E_g^1 basis states for the Hubbard molecule model for LnO_2. The spatially degenerate orbitals are indicated by the curved brackets. Only the states in red are used in the model.

O 2p E_g^3 states	Ce 5d E_g^1 states (↑)( )	(↓)( )	( )(↑)	( )(↓)
(↓)(↑↓)	ψ_3	ψ_7	ψ_{11}	ψ_{15}
(↑)(↑↓)	ψ_4	ψ_8	ψ_{12}	ψ_{16}
(↑↓)(↓)	ψ_5	ψ_9	ψ_{13}	ψ_{17}
(↑↓)(↑)	ψ_6	ψ_{10}	ψ_{14}	ψ_{18}

The Hubbard molecule model Hamiltonian for the 5d E_g orbitals in LnO_2 can be described using the following matrix for each of the two spatially independent wavefunctions ψ_1 and ψ_2.

$$\boldsymbol{A} = \begin{pmatrix} 0 & -t & -t \\ -t & U' & 0 \\ -t & 0 & U' \end{pmatrix}, \text{ where } \begin{pmatrix} 1 \\ 0 \\ 0 \end{pmatrix} = \psi_1 \text{ (or } \psi_2\text{)}, \begin{pmatrix} 0 \\ 1 \\ 0 \end{pmatrix} = \psi_3 \text{ (or } \psi_{13}\text{), and } \begin{pmatrix} 0 \\ 0 \\ 1 \end{pmatrix} = \psi_8 \text{ (or } \psi_{18}\text{)},$$

and t is the electron hopping term (interaction integral), which is analogous to off-diagonal matrix element, H_{ij}, (orbital interaction integral) in MO theory.

For each of the two spatially independent components of ψ_1, the eigenvalues for the model are 0 (singly degenerate) and $E_\pm = \frac{1}{2}\left(U' \pm \sqrt{U'^2 + 8t^2}\right)$. The eigenvectors we care about are $\psi_\pm$, which have energies $E_\pm$. The eigenvectors are determined by solving $\mathbf{A}$-$E_\pm\mathbf{I} = \mathbf{0}$. From the last 2 rows of $\mathbf{A}$, we get $a_2 = a_7$ (= $a_{12} = a_{17}$), and from the first row, we get $-E_\pm a_1 = t(a_2 + a_7) = 2ta_2$ or $a_2 = a_7 = -(E_\pm/2t)a_1$ taking $a_1 = 1$, we get $\psi_\pm = \psi_{1a} - (E_\pm/2t)[\psi_2 + \psi_7]$. Since $|L^4E_g^0\rangle$ is ψ_{1a} and $|L^3E_g^1\rangle$ is $(\psi_2 + \psi_7)/\sqrt{2}$, $\psi_\pm$ can be expressed as $\psi_\pm = |L^4E_g^0\rangle - (E_\pm/\sqrt{2}t)|L^3E_g^1\rangle$, which is not normalized. The normalized wavefunction is given by $\psi_\pm = N[|L^4E_g^0\rangle + \lambda|L^3E_g^1\rangle]$, where $N=1/\sqrt{1+\lambda^2}$, and $\lambda = -E_\pm/\sqrt{2}t$,

$E_\pm = \frac{1}{2}\left(U' \pm \sqrt{U'^2 + 8t^2}\right)$ $\psi_\pm = N[|L^4E_g^0\rangle + \lambda|L^3E_g^1\rangle]$, where $N=1/\sqrt{1+\lambda^2}$, and $\lambda = -E_\pm/\sqrt{2}t$.
$E_{MLCT} = \sqrt{U'^2 + 8t^2}$ and $n_f = 2(\lambda^2/N^2)$.

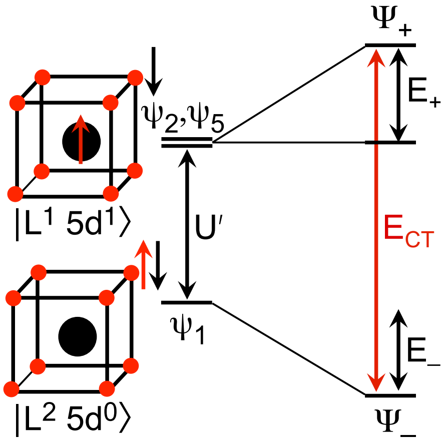

$\lvert L^1 5d^1\rangle$	$5d^1$ (↑)	$5d^1$ (↓)
L^1 (↓)	ψ_2	ψ_4
L^1 (↑)	ψ_3	ψ_5

ψ_1 is $\lvert L^2 5d^0\rangle$

Figure S2. HMM for one of the spatially degenerate 5d interactions in LnO_2 (left), and its basis states (right).

Hubbard molecule model for LnO_2: O 2p and Ln 5d, T_{2g} mixing

The ground state is $|L^6\,T_{2g}^0\rangle$, which corresponds to O 2p T_{2g}^6 and Ln 5d T_{2g}^0. This state is triply spatially degenerate and consists of wavefunctions ψ_1, ψ_2, and ψ_3. The energy of ψ_1, ψ_2, and ψ_3 is 0. The excited state that interacts with ψ_1, ψ_2, and ψ_3 is $|L^5T_{2g}^1\rangle$, which 36-fold degenerate and given by ψ_4 - ψ_{39}. The individual states are shown in Table S1. Only states in which the electron can hop from the occupied O 2p E_g orbitals into the Ln 5d E_g orbitals without changing sign can interact with the ground state: ψ_{1a} can interact with ψ_2 and ψ_7, and ψ_{1b} can interact with ψ_{12} and ψ_{17}, and ψ_{1c} can interact with ψ_{30} and ψ_{37}. The other states cannot interact with ψ_1 and are excluded from the model.

Table S1: T_{2g}^5 5d T_{2g}^1 basis states for the Hubbard molecule model of LnO_2. The spatially degenerate orbitals are indicated by the curved brackets. Only the states in red are used in the model.

O 2p T_{2g}^5 states	Ln 5d T_{2g}^1 states for $(d_{xz})(d_{yz})(d_{xy})$					
	(↑)( )( )	(↓)( )( )	( )(↑)( )	( )(↓)( )	( )( )(↑)	( )( )(↓)
(↓)(↑↓)(↑↓)	ψ_4	ψ_{10}	ψ_{16}	ψ_{22}	ψ_{28}	ψ_{34}
(↑)(↑↓)(↑↓)	ψ_5	ψ_{11}	ψ_{17}	ψ_{23}	ψ_{29}	ψ_{35}
(↑↓)(↓)(↑↓)	ψ_6	ψ_{12}	ψ_{18}	ψ_{24}	ψ_{30}	ψ_{36}
(↑↓)(↑)(↑↓)	ψ_7	ψ_{13}	ψ_{19}	ψ_{25}	ψ_{31}	ψ_{37}
(↑↓)(↑↓)(↓)	ψ_8	ψ_{14}	ψ_{20}	ψ_{26}	ψ_{32}	ψ_{38}
(↑↓)(↑↓)(↑)	ψ_9	ψ_{15}	ψ_{21}	ψ_{27}	ψ_{33}	ψ_{39}

The Hubbard molecule model Hamiltonian for the 5d T_{2g} orbitals in LnO_2 can be described using the following matrix for each of the three spatially independent components of ψ_1.

$\boldsymbol{A} = \begin{pmatrix} 0 & -t & -t \\ -t & U' & 0 \\ -t & 0 & U' \end{pmatrix}$, where $\begin{pmatrix}1\\0\\0\end{pmatrix} = \psi_1, \psi_2$, or ψ_3, $\begin{pmatrix}0\\1\\0\end{pmatrix} = \psi_4, \psi_{18}$, or ψ_{32}, and $\begin{pmatrix}0\\0\\1\end{pmatrix} = \psi_{11}, \psi_{25}$ or ψ_{39}

t is the electron hopping term (interaction integral), which is analogous to off-diagonal matrix element, H_{ij}, (orbital interaction integral) in MO theory.

For each of the three spatially independent components of $|L^6\,T_{2g}^0\rangle$, the eigenvalues for the model are 0 (singly degenerate) and $E_\pm = \frac{1}{2}\left(U' \pm \sqrt{U'^2 + 8t^2}\right)$. The eigenvectors we care about are $\psi_\pm$, which have energies $E_\pm$. The eigenvectors are determined by solving $\mathbf{A}$-$E_\pm\mathbf{I} = \mathbf{0}$. From the last 2 rows of $\mathbf{A}$, we get $a_4 = a_{11} = a_{18} = a_{25} = a_{32} = a_{39}$, and from the first row, we get $-E_\pm a_1 = t(a_4 + a_{11}) = 2ta_4$ or $a_4 = a_{11} = -(E_\pm/2t)a_1$ taking $a_1 = 1$, we get $\psi_\pm = \psi_1 - (E_\pm/2t)[\psi_4 + \psi_9]$. Since $|L^6T_{2g}^0\rangle$ is ψ_1 and $|L^5T_{2g}^1\rangle$ is $(\psi_4 + \psi_{11})/\sqrt{2}$, so $\psi_\pm = N[|L^6T_{2g}^0\rangle - (E_\pm/\sqrt{2}t)|L^5T_{2g}^1\rangle]$, where $N=1/\sqrt{1+\lambda^2}$, and $\lambda = -E_\pm/\sqrt{2}t$:

$E_\pm = \frac{1}{2}\left(U' \pm \sqrt{U'^2 + 8t^2}\right)$ and $\psi_\pm = N[|L^6E_g^0\rangle + \lambda|L^5E_g^1\rangle]$, where $N=1/\sqrt{1+\lambda^2}$, and $\lambda = -E_\pm/\sqrt{2}t$. $E_{MLCT} = \sqrt{U'^2 + 8t^2}$ and $n_f = 3(\lambda^2/N^2)$.

HMM to second order for CeO_2 4f interaction

The energies can be determined to second order by solving |**A**-E**S**| = 0, where **A** is Hamiltonian for the HMM and **S** are the overlap integrals. S_{ii} is equal to 1 and there is only one unique value of S_{ij}, which can be shortened to S. For the CeO_2 4f interaction, the determinant is given below

$$\begin{vmatrix} -E & 0 & -t-ES \\ 0 & -E & -t-ES \\ -t-ES & -t-ES & U'-E \end{vmatrix} = 0 \text{ with } t = -S(2E_F + U'), \text{ and } E_F \text{ -7.4 eV}$$

The eigenvalues are 0 and $E_{\pm} = \frac{1}{2(1-2S^2)}\left(U' + 4St \pm \sqrt{U'^2 + 8t^2 + 8U'St}\right)$. The eigenvectors of interest are $\psi_{\pm}$, which have energies $E_{\pm}$. The eigenvectors are determined from **A**-$E_{\pm}$**S** = **0**. From the first 2 rows of **A**, $a_1 = a_4$ and $E_{\pm}a_1=(-t- E_{\pm}S)a_5$ or $a_5 = -E_{\pm}/(t+ E_{\pm}S)\, a_1$, where a_i is the coefficient of ψ_i. Using $a_1 = 1$, the unnormalized wavefunction is $\psi_{\pm} = \psi_1 + \psi_4 - E_{\pm}/(t+ E_{\pm}S)\psi_5$. The normalization constant, N, is $1/\sqrt{2 + (E_{\pm}/(t + E_{\pm}S))^2}$. The $\psi_{\pm}$ can be simplified by dividing by $\sqrt{2}$ to give $\psi_{\pm} = N\left[(\psi_1 + \psi_4)/\sqrt{2} - \left(E_{\pm}/\sqrt{2}\,(t + E_{\pm}S)\right)\psi_5\right]$, with $N=1/\sqrt{1 + \left(E_{\pm}/\sqrt{2}\,(t + E_{\pm}S)\right)^2}$. Finally, $(\psi_1 + \psi_4)/\sqrt{2}$ can be more conveniently represented as $|L^1 4f^1\rangle$ and ψ_5 by $|L^2 4f^0\rangle$, to give the following:

$E_{\pm} = \frac{1}{2(1-2S^2)}\left(U' + 4St \pm \sqrt{U'^2 + 8t^2 + 8U'St}\right)$ and $\psi_{\pm} = N(|L^1\, 4f^1\rangle + \lambda|L^2\, 4f^0\rangle)$ where $N=1/\sqrt{1 + \lambda^2}$, $\lambda_{\square} = -E_{\pm}/\left[\sqrt{2}(t + E_{\pm}S)\right]$.

$E_{CT} = \frac{1}{1-2S^2}\left(\sqrt{U'^2 + 8t^2 + 8U'St}\right)$ and $n_f = 1/N^2$. $t = -S(2E_F + U')$ with E_F -7.4 eV.

HMM to second order for PrO_2 4f interactions.

The model for PrO_2 is the same as for CeO_2.

$E_{\pm} = \frac{1}{2(1-2S^2)}\left(U' + 4St \pm \sqrt{U'^2 + 8t^2 + 8U'St}\right)$ and $\psi_{\pm}$ = N(|L^1 $4f^1$⟩ + λ|L^2 $4f^0$⟩) where N=$1/\sqrt{1+\lambda^2}$, $\lambda_{□}$ = $-E_{\pm}/\left[\sqrt{2}(\text{t} + E_{\pm}\text{S})\right]$ .E_{MLCT} =$\frac{1}{1-2S^2}\left(\sqrt{U'^2 + 8t^2 + 8U'St}\right)$ and n_f = 1/N^2. Using $t = -S(2E_F + U')$ with E_F -7.4 eV.

HMM to second order for TbO_2 4f interactions.

The HMM for TbO_2 can be expressed as doubly degenerate (spin degenerate) with

$\boldsymbol{A} = \begin{pmatrix} 0 & -t \\ -t & U' \end{pmatrix}$, where $\begin{pmatrix} 1 \\ 0 \end{pmatrix}$ = $\psi_{□}$ or ψ_2, $\begin{pmatrix} 0 \\ 1 \end{pmatrix}$ = ψ_3 or ψ_4

Solving |**A**-E**S**| = 0 gives $E_{\pm} = \frac{1}{2(1-S^2)}\left(U' + 2St \pm \sqrt{U'^2 + 4t^2 + 4U'St}\right)$ and $\psi_{\pm}$ = N(|L^2 $4f^0$⟩ + λ|L^1 $4f^1$⟩) where N=$1/\sqrt{1+\lambda^2}$, $\lambda_{□}$ = $-E_{\pm}/[(\text{t} + E_{\pm}\text{S})]$. E_{CT} =$\frac{1}{1-S^2}\left(\sqrt{U'^2 + 4t^2 + 4U'St}\right)$ and n_f = 2$\lambda^{□}$/N^2. $t = -S(2E_F + U')$ with E_F -7.4 eV.

HMM to second order for LnO_2: O 2p and Ln 5d interactions

The HMMs for the T_{2g} and E_g interactions are essentially the same apart from their spatial degeneracy. In both cases, the interaction can be broken into non-degenerate cases (e.g., the double degenerate E_g interaction can be divided into the d_{z^2} and $d_{x^2-y^2}$ interactions). For each of the non-degenerate cases, the HMM can be expressed as

$\boldsymbol{A} = \begin{pmatrix} 0 & -t & -t \\ -t & U' & 0 \\ -t & 0 & U' \end{pmatrix}$, where $\begin{pmatrix} 1 \\ 0 \\ 0 \end{pmatrix} = |L^2\ 5d^0\rangle$ and $\begin{pmatrix} 0 \\ 1 \\ 0 \end{pmatrix}$ and $\begin{pmatrix} 0 \\ 0 \\ 1 \end{pmatrix}$ are $|L^1\ 5d^1\rangle$.

Solving |**A**-E**S**| = 0 gives the same solution as for CeO_2 4f interactions. $E_{\pm} = \frac{1}{2(1-2S^2)}\left(U' + 4St \pm \sqrt{U'^2 + 8t^2 + 8U'St}\right)$ and $\psi_{\pm} = N(|L^1\ 4f^1\rangle + \lambda|L^2\ 4f^0\rangle)$ where $N = 1/\sqrt{1+\lambda^2}$, $\lambda_{□} = -E_{\pm}/[\sqrt{2}(t + E_{\pm}S)]$,

$E_{CT} = \frac{1}{1-2S^2}\left(\sqrt{U'^2 + 8t^2 + 8U'St}\right)$ and $n_f = n_{deg}\lambda^2/N^2$, where n_{deg} is the degree of degeneracy. $t = -S(2E_F + U')$ with E_F -7.4 eV.

Experimental details

Magnetic susceptibility measurements. In an argon filled glovebox, samples were loaded into 3 mm OD quartz tubes by sandwiching them between two plugs of quartz wool. The samples were compressed into a pellet by squeezing them between two quartz rods. The quartz rods were removed, and the ends of the tube were capped by inserting them into septa for 7 mm tubing. The capped tube was removed from the glovebox. The center of the tube was wrapped with a Kimwipe, saturated with liquid nitrogen, and sealed with a propane/oxygen torch. Variable temperature magnetization data were recorded at 1 T, 2 T, and 4 T using a Quantum Designs MPMS SQUID magnetometer.
Variable temperature magnetization was corrected for the diamagnetism of the quartz wool using Pascal's constants for covalent compounds, $\chi_{QW} = 3.7 \times 10^{-7}$ emu g^{-1} (no correction for the diamagnetism of the quartz tube is needed as it never leaves the SQUID coils). Molar susceptibility was calculated using the following equation:

$$\chi_{mol} = \frac{(\text{molecular weight})}{(\text{sample mass})}\left[\frac{(M_{meas} - M_{ferro})}{H} - \chi_{QW}\right] - \chi_{dia}$$

Where χ_{mol} is the molar susceptibility, M_{meas} is the measured magnetization, M_{ferro} is the magnetization of the ferromagnetic impurity, which is temperature and field- independent; χ_{QW} is the contribution to the susceptibility due to the quartz wool, χ_{dia} is the diamagnetic correction determined using Pascal's constants, and H is the applied field.
Two ferromagnetic impurities are commonly encountered in laboratory samples, ferrous metals and magnetite or other ferrites from the oxide coating on stainless steel lab equipment. Of these, magnetite is far more likely to be encountered. In general, the magnetization of ferromagnets is temperature independent below the Curie temperature, which is 860 K for magnetite, so magnetization of the impurity is temperature independent for this experiment. The magnetization of magnetite reaches saturation at approximately 0.2 T, above which the magnetization is ~90 emu/g. Below this field, the magnetization of magnetite is roughly linear with applied field. Based on the assumption that the impurity is magnetite or a related ferrite resulting from the abrasion of stainless steel lab equipment, the data were corrected for a temperature and field independent ferromagnetic impurity. M_{ferro} was allowed to vary to minimize the least squares difference between χ_{mol} at different fields. Variable temperature magnetic susceptibility data including before and after the correction for ferromagnetic impurities are included in the SI.

Diffuse reflectance (DR) measurements. DR spectra were obtained with an Ocean Optics T3000 spectrometer equipped with a diffuse reflectance probe. Samples were smeared onto a glass microscope slide covered with several layers of poly-tetrafluoroethylene (PTFE) tape. The blank spectrum was obtained from the PTFE-covered microscope slide prior to taking the data on the compound. Reflectance data were converted to $F(R_\infty)$ using the Kubelka-Munk transform.[1] The DR spectra were normalized by setting the lowest absorbance of the spectrum to zero (Figures S1 and S2).

Charge-transfer band gap energy (E_{BG}) determination. Tauc plots were generated assuming that the O 2p to Ln 4f and 5d transitions are allowed, direct transitions.[1-3] The band gap was determined using the approach described by Makula et al.[1] Briefly, the data are plotted as $[F(R_\infty)\cdot h\upsilon]^2$ vs hυ (DR spectra) or $(\alpha\cdot h\upsilon)^2$ vs hυ (XANES spectra), where α is the absorbance. The regions below and above the transition are fit to straight lines. The intercept of these lines is

the band gap. In the case of the O 2p to 4f transitions in the DR spectra of LnO_2, the intercept with the x-axis was used instead of fitting the region before the transition. To determine the CeO_2 O 2p to Ce 5d band gaps, the CeO_2 absorption spectrum reported by Niwano et al. was digitized using the program UN-SCAN-IT, and the band gaps were determined using the approach described by Makula et al.[1, 4-5] The uncertainty in Tauc plot determinations of band gaps is typically reported as 0.03 eV. Based on work on CeO_2 thin films, we assume the uncertainty is slightly larger, 0.05 eV, for the band gap determined from the UV-Visible DR data (the numerical error from fitting the Tauc plots is smaller).[6]

Least Squares fitting and uncertainty analysis. Least squares fitting of the XANES pre-edge energies to the charge transfer band gap energies with errors in both dimensions was performed as described in "Numerical Recipes."[7] Uncertainties in the modeled parameters, σ_f, were determined using $\sigma_f^2 = \sum_i \left(\frac{\partial f}{\partial x_i}\right)^2 \sigma_{x_i}^2$, where σ_{xi} is the uncertainty of measured property x_i, and the derivatives were determined numerically.

References

1. Makuła, P.; Pacia, M.; Macyk, W., How To Correctly Determine the Band Gap Energy of Modified Semiconductor Photocatalysts Based on UV–Vis Spectra. *The Journal of Physical Chemistry Letters* **2018,** *9* (23), 6814-6817.
2. Sun, L.; Xiao, W.; Hao, X.; Meng, Q.; Zhou, M., A first-principles study on the structural, thermal and electronic properties of cerium oxides by using different functionals. *Electronic Structure* **2018,** *1* (1), 015003.
3. Tauc, J.; Grigorovici, R.; Vancu, A., Optical Properties and Electronic Structure of Amorphous Germanium. *physica status solidi (b)* **1966,** *15* (2), 627-637.
4. Silk Scientific Corporation *UN-SCAN-IT 5.2*, 2012.
5. Niwano, M.; Sato, S.; Koide, T.; Shidara, T.; Fujimori, A.; Fukutani, H.; Shin, S.; Ishigame, M., Optical Properties of CeO2 Crystal in the Photon Energy Range of 2.5–40 eV. *Journal of the Physical Society of Japan* **1988,** *57* (4), 1489-1496.
6. Chiu, F.-C.; Lai, C.-M., Optical and electrical characterizations of cerium oxide thin films. *Journal of Physics D: Applied Physics* **2010,** *43* (7), 075104.
7. Press, W. H.; Teukolsky, S. A.; Vetterling, W. T.; Flannery, B. P., *Numerical Recipes in FORTRAN The Art of Scientific Computing, Second Ed.* Cambridge University Press: New York, 1992.

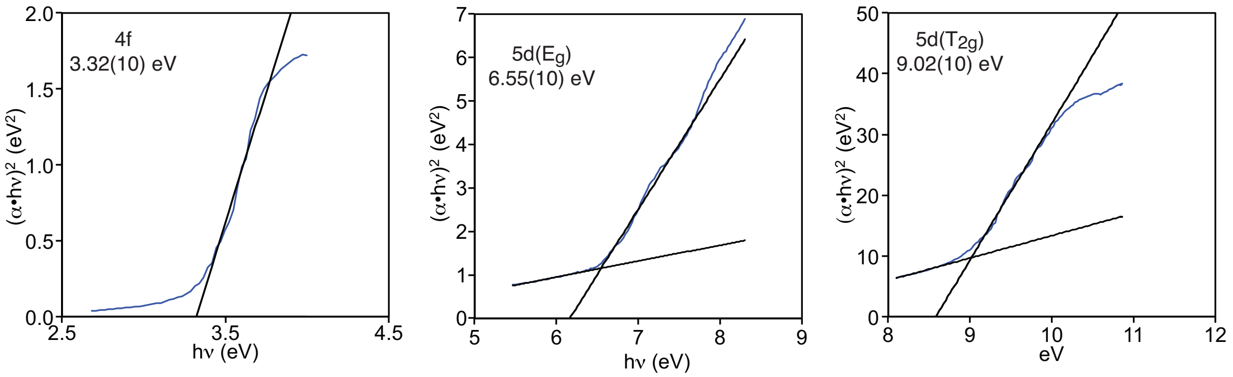

Figure S3. Tauc plots for CeO_2 from the data published by Niwano, et al.[5] Band gap given in the upper left corner of each plot.

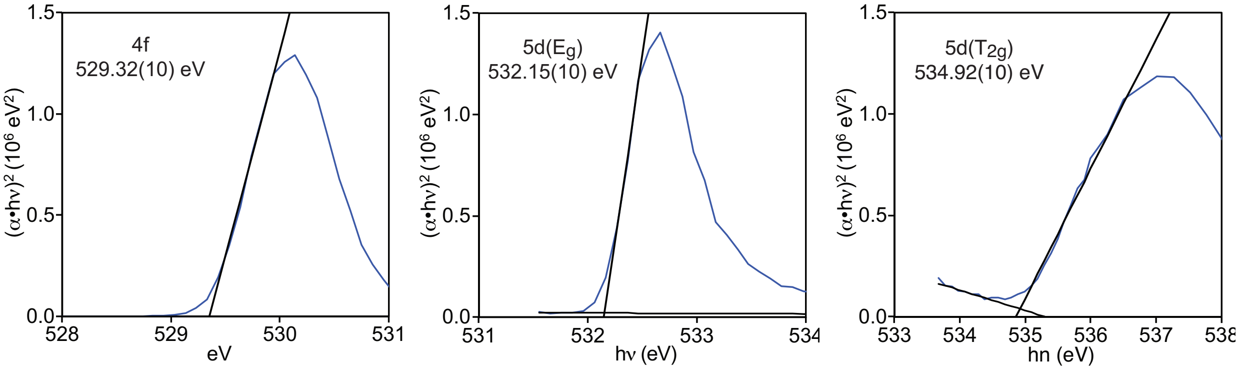

Figure S4. Tauc plots for CeO_2 O K-edge XANES pre-edge peaks. Band gap given in the upper left corner of each plot.

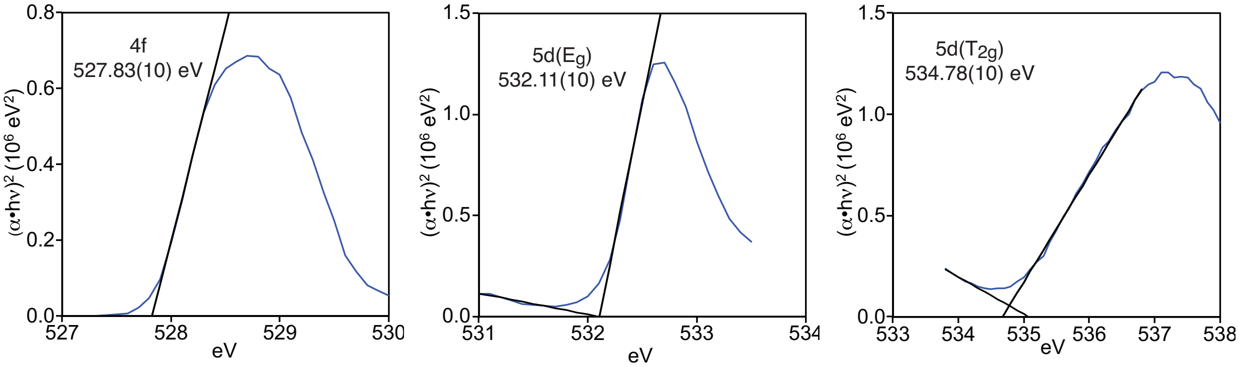

Figure S5. Tauc plots for PrO_2 O K-edge XANES pre-edge peaks. Band gap given in the upper left corner of each plot.

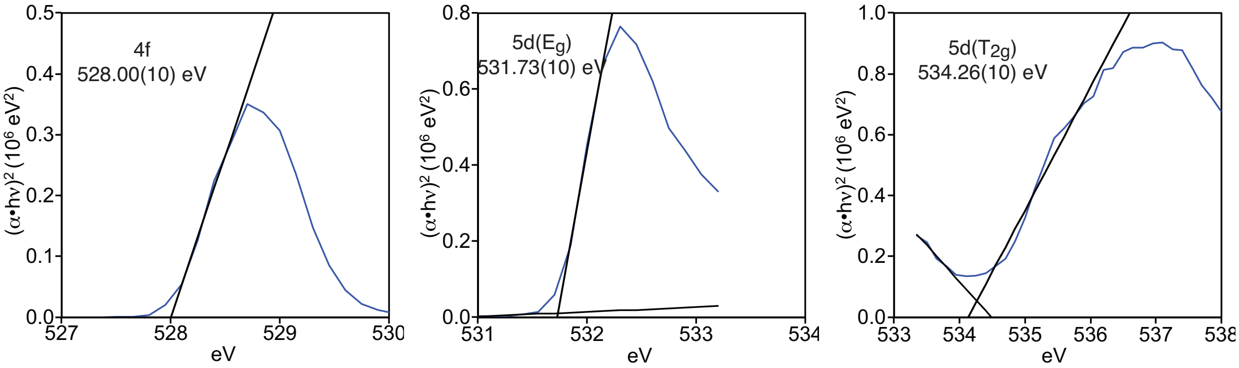

Figure S6. Tauc plots for TbO_2 O K-edge XANES pre-edge peaks. Band gap given in the upper left corner of each plot.

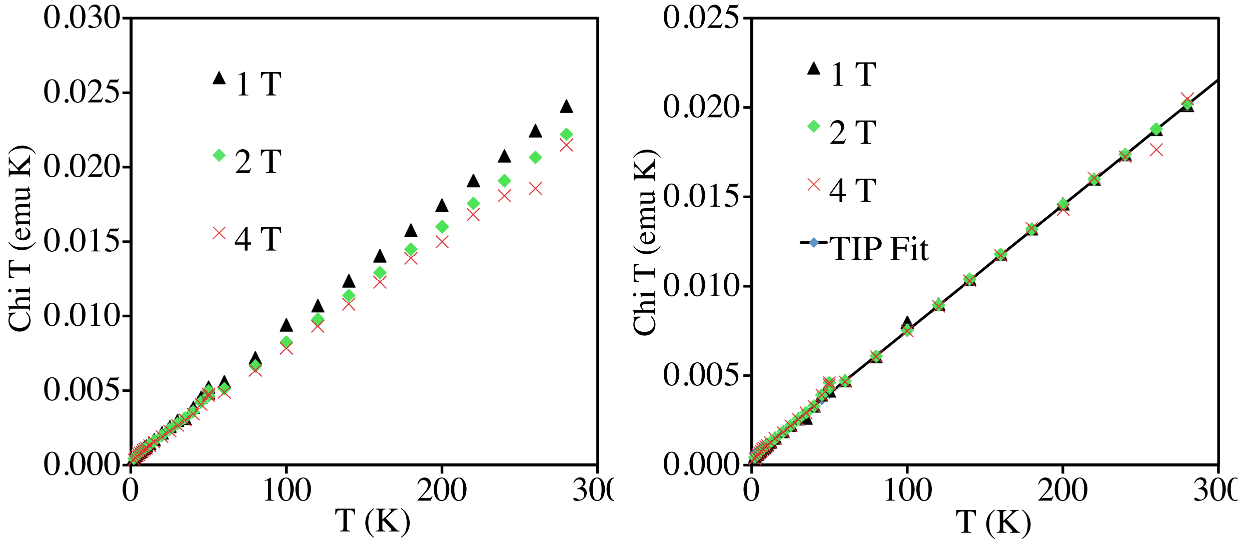

Figure S7. Magnetic Susceptibility of CeO_2 from Aldrich, dried under vacuum for 48 hr. Data are corrected for underlying diamagnetism using Pascal's constants. (left) Data before correction for a ferromagnetic impurity. (right) Data corrected for a ferromagnetic impurity.

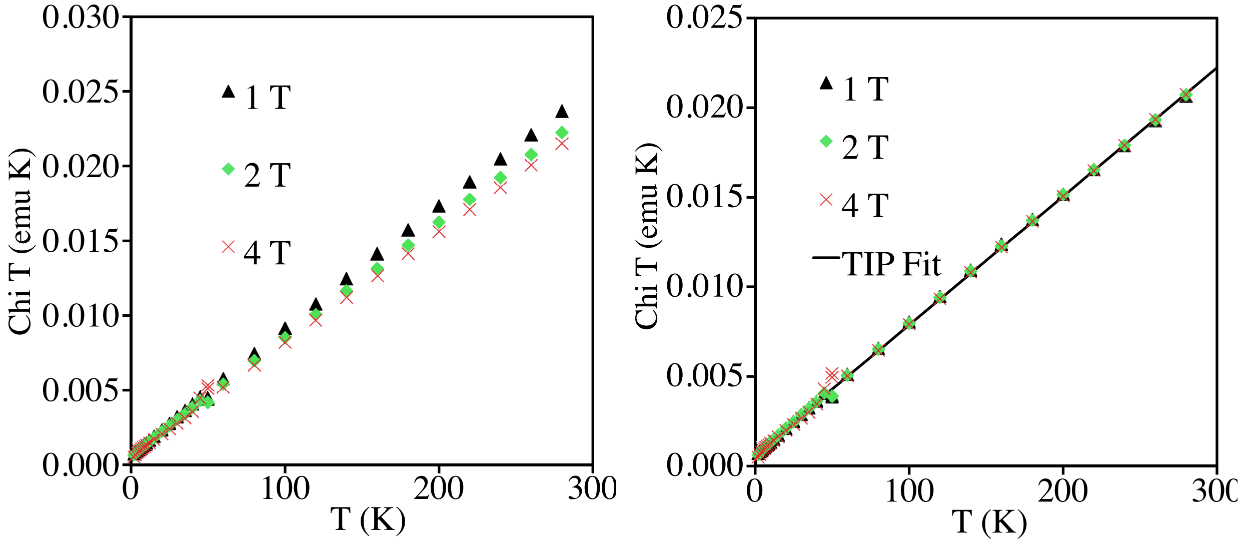

Figure S8. Magnetic Susceptibility of CeO_2 from Strem, dried under vacuum for 48 hr. Data are corrected for underlying diamagnetism using Pascal's constants. (left) Data before correction for a ferromagnetic impurity. (right) Data corrected for a ferromagnetic impurity.
